# A pyramid-like model for heartbeat classification from ECG recordings

**DOI:** 10.1371/journal.pone.0206593

**Published:** 2018-11-14

**Authors:** Jinyuan He, Le Sun, Jia Rong, Hua Wang, Yanchun Zhang

**Affiliations:** 1 Institute of Sustainable Industries & Liveable Cities, VU Research, Victoria University, Melbourne, VIC, Australia; 2 School of computer and software, Nanjing University of Information Science and Technology, Nanjing, Jiangsu, China; Universita degli Studi di Pisa, ITALY

## Abstract

Heartbeat classification is an important step in the early-stage detection of cardiac arrhythmia, which has been identified as a type of cardiovascular diseases (CVDs) affecting millions of people around the world. The current progress on heartbeat classification from ECG recordings is facing a challenge to achieve high classification sensitivity on disease heartbeats with a satisfied overall accuracy. Most of the work take individual heartbeats as independent data samples in processing. Furthermore, the use of a static feature set for classification of all types of heartbeats often causes distractions when identifying supraventricular (S) ectopic beats. In this work, a pyramid-like model is proposed to improve the performance of heartbeat classification. The model distinguishes the classification of *normal* and *S* beats and takes advantage of the neighbor-related information to assist identification of *S* bests. The proposed model was evaluated on the benchmark *MIT-BIH-AR* database and the *St. Petersburg Institute of Cardiological Technics*(INCART) database for generalization performance measurement. The results reported prove that the proposed pyramid-like model exhibits higher performance than the state-of-the-art rivals in the identification of disease heartbeats as well as maintains a reasonable overall classification accuracy.

## Introduction

An electrocardiogram (ECG) is a recording of the electrical activity of the heart over a period of time. It provides a noninvasive and inexpensive way for studying the heart. Heartbeat classification is one of the important fields in ECG analysis. The Association for Advancement of Medical Instrumentation (AAMI) categorized heartbeats into 5 classes: Normal(*N*), Supraventricular (*S*) ectopic, Ventricular (*V*) ectopic, Fusion (*F*) and Unknown (*Q*) beats [1]. Heartbeat classification is an essential step toward identifying arrhythmias. Arrhythmias affect the body by impacting heart’s ability to pump blood. Critically, arrhythmias can be divided as life-threatening and non-life-threatening ones [2]. For example, ventricular fibrillation and tachycardia are life-threatening arrhythmias, which are fatal and require medical attention immediately. Non-life-threatening arrhythmias, such as atrial fibrillation, just present a chronic health threat to patients, but special care is still needed to avoid further deterioration of heart function.

Although to perform an electrocardiography test is simple, the manual interpretation of ECG recordings could be time-consuming and error-prone, especially for the long-term ECG recordings. Hence, an intelligent approach on automatic heartbeat classification from ECG recordings is highly demanded, which would be of great assistance for clinicians in heart diseases diagnosis.

Many research attempts have been made to address the heartbeat classification problem. The current process has difficulties in guarantying a high detection sensitivity of disease heartbeats as well as maintaining a good overall classification accuracy. Most of the existing work take heartbeats as mutual-independent data samples, with no connections to their predecessors or successors [2–6]. Therefore, the neighbor-related information is ignored in their classification process. In addition, the use of a single static feature set to classify all types of heartbeats together may cause high misclassification on *S* beats in particular. A number of factors need to be further considered in classification: (1) ECG recordings are imbalanced and usually dominated by the *N* beats; (2) Some shape-related features must be included to distinguish the *V* beats from the *N* beats for they have different *QRS* complexes; (3) The *N* and *S* beats are similar in *QRS* complex morphology, but the *S* beats have a fast heart rhythm. In other words, the existence of the shape-related features makes a *S* beat be easily misidentified as a *N* beat. In this study, we aim to propose a pyramid-like model to solve these problems and improve the heartbeat classification performance.

## Related work

The related studies in heartbeat classification from ECG recordings are reviewed in this section. Besides, we introduce two feature extraction techniques—the Higher-order statistics and the Discrete wavelet transformation. The *Earth mover’s distance* (EMD) is also discussed for measuring the dissimilarity of two multi-dimensional distributions.

### Literature review

Many machine-learning approaches have been proposed for automatic heartbeat classification since last two decades. The variety of classification performance among these approaches are primarily the features and the classifiers used.

The features used to represent a heartbeat are usually extracted from cardiac rhythm or time/frequency domains, in which the *RR*-Interval is reported as one of the most widely used feature [2, 3, 7–10]. *RR*-Interval holds indispensable information about heart rhythms and has capacity to discriminate the disease heartbeats from the normal ones. Other features, such as the *higher order statistics* (HOS) [7, 11], *wavelet coefficients* [12–17], *morphological amplitudes* [2, 18], *signal energy* [17], and *random projection features* [19, 20], can also be commonly found in the literature. As irrelevant features could cause negative impacts to the classification performance and decrease the generalization power, different feature selection techniques have been applied to clear up the noise and reduce the feature dimension, such as the *floating sequential search* [4] and the *weighted linear discriminant model with a forward-backward search strategy* [21].

Regarding the classifiers, the *support vector machine* (SVM) [8, 20, 22–24], *nearest neighbors* (NN) [25, 26], *artificial neural networks* (ANN) [13, 27], *optimum-path forest* (OPF) [28], *linear discriminants*(LD) [3], *conditional random field* [11], and *reservoir computing with logistic regression* [29] are common choices for the heartbeat classification problem. However, using a single classifier can bias the classification and lead to a relatively low generalization performance. Some ensemble methods, such as random forest [7] and ensemble of SVM [20], have been employed to remedy the disadvantages.

Although some promising results have been achieved, the current methods on heartbeat classification still have some problems. The associations among heartbeats are often ignored in existing classification process. All types of heartbeats are presented using a same set of static features. This could limit the classification performance and possibly lead to a failure in identification of *S* beats. Therefore, heartbeat classification is seeking for a solution to provide high accuracy.

### Higher-order statistics

The *higher-order statistics* (HOS) methods are commonly used to estimate signal shape. They contain both amplitude and phase information of non-Gaussian linear processes and high immunity to the Gaussian background noise in comparison to the lower-order statistics [30]. In this work, we counted the *skewness* (3rd order statistics) and the *kurtosis* (4th order statistics) into our feature set.

The *skewness* measures the symmetry of a distribution. The *kurtosis* denotes whether the distribution is heavy-tailed or light-tailed, as compared to the normal distribution. For an input signal, assume *X*_1…,*N*_ denotes all the data samples, $\bar{X}$ is the mean and *s* is the standard deviation, the *skewness* and *kurtosis* can be defined respectively as below.

$$\begin{array}{c} Skewness=\frac{\sum_{i=1}^{N}{\left(X_{i}-\bar{X}\right)}^{3}/N}{s^{3}} \end{array}$$ (1)

$$\begin{array}{c} Kurtosis=\frac{\sum_{i=1}^{N}{\left(X_{i}-\bar{X}\right)}^{4}/N}{s^{4}} \end{array}$$ (2)

### Discrete wavelet transform

**Fig 1:**
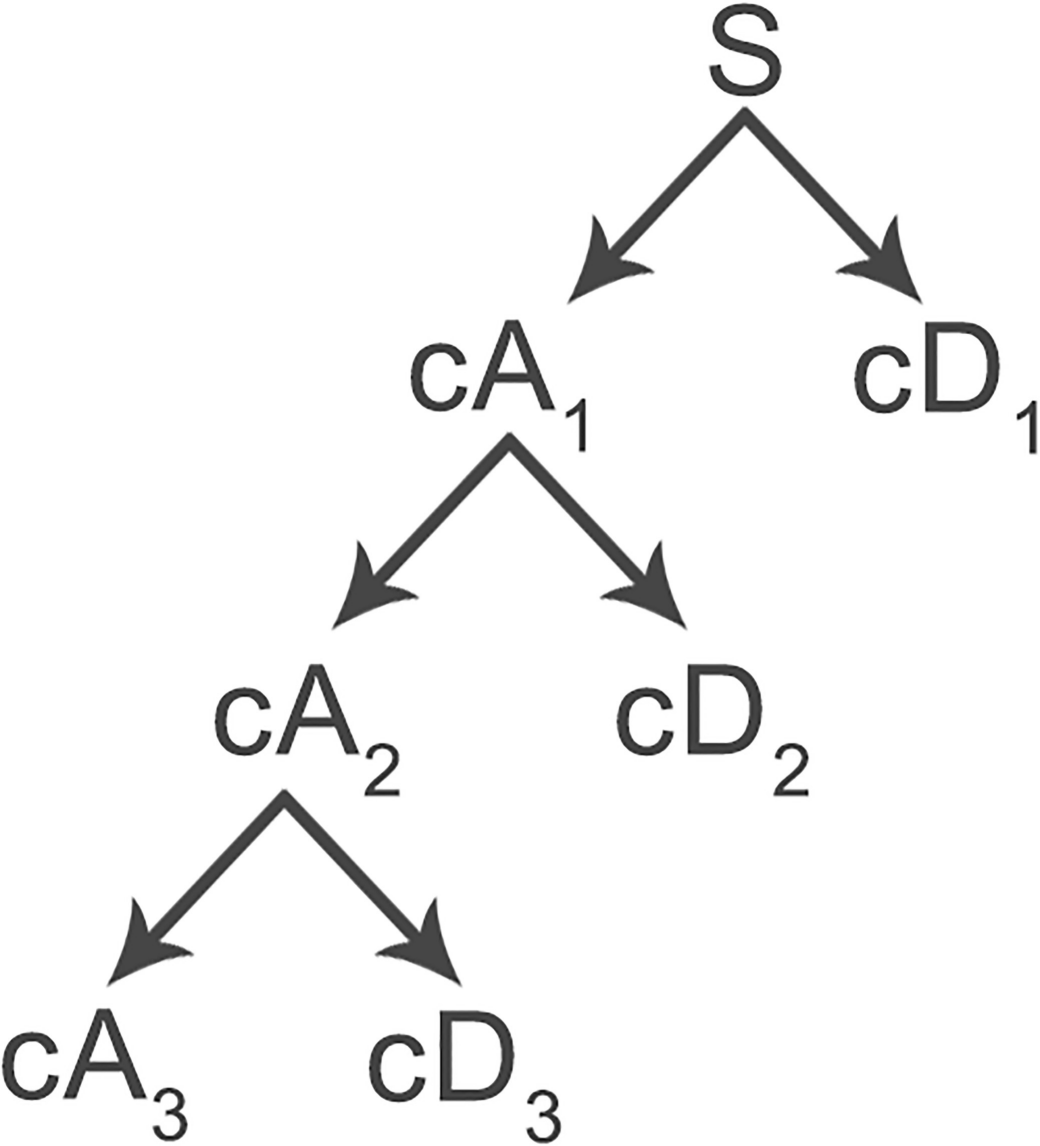
A demonstration of discrete wavelet decomposition. *cD*_*x*_ denote the wavelet coefficients of coarse approximation and detail information at *x* level, respectively.

The *discrete wavelet transform* (DWT) provides a time-frequency representation of a signal, which is widely used in data compression, noise reduction and multi-frequency-bands signal analysis. The DWT iteratively decomposes a signal to different frequency bands with a scaling function and a wavelet function. The high-frequency component provides the detail information; while the low-frequency components is a coarse approximation of the upper-level signal. Each component is represented by a collection of wavelet coefficients, which is obtained by the inner products of mother wavelet function and the upper-level signal. Fig 1 presents the whole decomposition process. Only the low-frequency components are decomposed.

The choice of the mother wavelet function is the key of the *discrete wavelet transform*, which heavily depends on applications. In term of noise reduction on raw ECG signals, we use the Daubechies-4 wavelet for its good orthogonality and short vanishing moment. For morphology features extraction, the Haar wavelet is chosen because of its simplicity. Besides, it has been demonstrated as the ideal wavelet for short time signal analysis [17]. The Haar function can be represented as

$$\begin{array}{c} \psi \left(t\right)=\{\begin{array}{ccc} 1 & & 0\leq t<1/2,\\ -1 & & 1/2\leq t<1,\\ 0 & & otherwise. \end{array}, \end{array}$$ (3)

and its corresponding scaling function is

$$\begin{array}{c} \phi \left(t\right)=\{\begin{array}{ccc} 1 & & 0\leq t<1,\\ 0 & & otherwise. \end{array}, \end{array}$$ (4)

where *t* denotes sample values.

### Earth mover’s distance

The *Earth mover’s distance* (EMD) is a metric of dissimilarity between two multi-dimensional distributions [31]. A distribution can be represented by a set of clusters. Such a representation is called the *signature* of the distribution. Data points from a distribution are grouped into a set of clusters, with each cluster denoted by its mean (or mode) and the fraction of the distribution that belongs to the cluster. Thus, one cluster can be regarded as a single feature in a signature. The distance between the features is called the *ground distance*. Signatures could be different in length. For example, simple distributions have shorter signatures than the complex ones.

The *Earth mover’s distance* can be formulated and solved as a *transportation problem* [32]. Assume that there is a signature *P* with *m* cluster:

$$\begin{array}{c} P=\{(p_{1},w_{p1}),\ldots ,(p_{m},w_{pm})\}, \end{array}$$ (5)

and a signature *Q* with *n* cluster:

$$\begin{array}{c} Q=\{(q_{1},w_{q1}),\ldots ,(q_{n},w_{qn})\}, \end{array}$$ (6)

where *p* and *q* are the cluster representatives (mean or mode), and *w* denotes the cluster weight.

Let *D* = [*d*_[_*i*, *j*]] be the ground distance between *p*_*i*_ and *q*_*j*_ and *F* = [*f*_*i*,*j*_] be the flow between *p*_*i*_ and *q*_*j*_. The optimal *F* is obtained by minimizing the overall work:

$$\begin{array}{c} W=\sum_{i=1}^{m}\sum_{j=1}^{n}f_{i,j}d_{i,j}, \end{array}$$ (7)

subject to the following constrains:

$$\begin{array}{c} 0\leq f_{i,j},1\leq i\leq m,1\leq j\leq n, \end{array}$$ (8)

$$\begin{array}{c} \sum_{j=1}^{n}f_{i,j}\leq w_{pi},1\leq i\leq m, \end{array}$$ (9)

$$\begin{array}{c} \sum_{i=1}^{m}f_{i,j}\leq w_{qj},1\leq j\;leqn, \end{array}$$ (10)

$$\begin{array}{c} \sum_{i=1}^{m}\sum_{j=1}^{n}f_{i,j}=min\{\sum_{i=1}^{m}w_{pi},\sum_{j=1}^{n}w_{qj}\} \end{array}$$ (11)

The *Earth mover’s distance* is defined as the work normalized by the total flow:

$$\begin{array}{c} EMD\left(P,Q\right)=\frac{\sum_{i=1}^{m}\sum_{j=1}^{n}f_{i,j}d_{i,j}}{\sum_{i=1}^{m}\sum_{j=1}^{n}f_{i,j}} \end{array}$$ (12)

## Methodology

This section presents the proposed methodology. Firstly, we introduce the preprocessing method. Then we discuss the appropriate features for heartbeat classification. After that, we present the pyramid-like model in detail.

### Preprocessing

**Fig 2:**
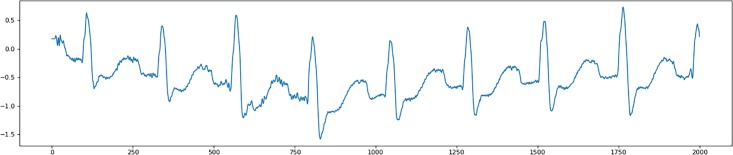
A sample ECG recording with Gaussian white noise and baseline wanders.

The raw ECG signals always come with Gaussian white noise and baseline wanders. The baseline wanders is the effect that the base axis (X-axis) of individual heartbeats appear to move up or down rather than being straight all the time, as shown in Fig 2. In order to avoid propagation of the negative impact of these two problems to the classification stage, an effective method for cleaning up the ECG recordings is indispensable.

To correct the baseline wanders, each ECG recording is processed with a 200-ms width median filter followed by a 600-ms median filter to obtain the signal baseline, which is then subtracted from the raw ECG signal to get the baseline corrected data. Then, a discrete wavelet transform is applied to remove the Gaussian white noise. The baseline corrected recordings are decomposed to different frequency bands with various resolutions. We select the *Daubechies-4* as the mother wavelet function because its short vanishing moment is ideal for analyzing signals like ECG with sudden changes. The coefficients of detail information (*cD*_*x*_) in each frequency band are then processed by a high-pass filter with a threshold value

$$\begin{array}{c} T=\sqrt{2*log(n)}, \end{array}$$ (13)

where *n* indicates the length of the input signal. The coefficients that failed by the filter are set to zero. Finally, the clean recordings are obtained by employing inverse discrete wavelet transform on the coefficients.

After noise reduction, The ECG recordings are segmented to individual heartbeats using the *R* locations provided by the databases. For each *R* peak, 90 samples (250-ms) before *R* peak and 144 samples (400-ms) after *R* peak are taken to represent a heartbeat. This is long enough to catch the samples representing the re-polarization of ventricular and short enough to exclude the neighbor heartbeats [7].

### Feature extraction

**Fig 3:**
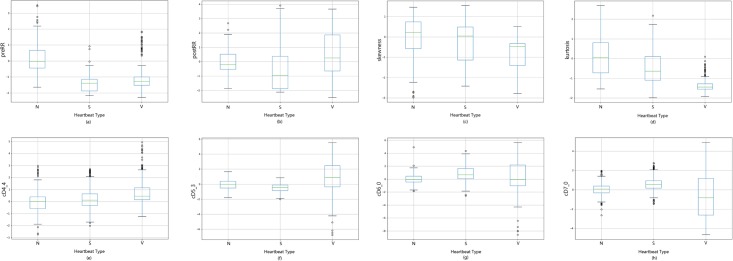
Boxplots for the extracted features of ECG signals.

Three types of features are used to characterize a heartbeat in this work: *RR*-interval, HOS and *wavelet coefficients*. Table 1 summarizes the statistics of these features and gives their p-values among the *N*, *S* and *V* beats. Fig 3 gives a visual demonstration on the feature significance via boxplots. The boxplot of each wavelet coefficient can be found in the S2 File.

**Table 1:** Feature statistics and the corresponding p-values between heartbeat classes.

Feature	Statistics (mean ± std)			P-values		
	N	S	V	N − S	N − V	S − V
preRR	[-0.81, 1.17]	[-1.98, -0.79]	[-1.86, -0.33]	2.16e−58	2.31e−38	4.27e−05
postRR	[-0.88, 0.89]	[-2.02, 0.79]	[-1.17, 1.99]	1.63e−07	1.94e−03	2.69e−11
skewness	[-0.99, 1.01]	[-1.36, 0.58]	[-1.63, -0.13]	8.48e−05	2.71e−21	2.33e−08
kurtosis	[-0.91, 1.09]	[-1.29, 0.35]	[-1.63, -0.91]	1.61e−09	5.42e−54	2.05e−30
cD4_0	[-0.82, 0.98]	[-0.95, 1.98]	[-2.38, 1.88]	3.47e−04	4.53e−02	3.33e−05
cD4_1	[-0.98, 0.7]	[-0.67, 1.57]	[-1.0, 2.29]	4.24e−09	3.15e−09	1.65e−01
cD4_2	[-0.98, 1.01]	[-0.6, 1.44]	[-1.2, 1.24]	7.40e−05	9.42e−01	4.77e−04
cD4_3	[-0.77, 0.84]	[-0.86, 0.35]	[-2.52, 0.83]	6.81e−05	7.24e−11	3.35e−06
cD4_4	[-0.54, 0.96]	[-0.32, 0.74]	[-2.71, 0.62]	9.42e−01	4.76e−20	1.08e−21
cD4_5	[-1.0, 0.96]	[-0.76, 1.23]	[-0.55, 1.83]	9.84e−03	3.52e−09	2.80e−04
cD4_6	[-0.97, 1.15]	[-1.37, 0.35]	[-1.25, 1.32]	1.35e−09	6.50e−01	8.73e−07
cD4_7	[-1.03, 0.74]	[-1.57, 0.84]	[-1.18, 3.22]	3.94e−02	1.76e−11	6.51e−14
cD4_8	[-0.79, 0.86]	[-1.09, 0.75]	[-1.97, 2.47]	1.86e−02	2.05e−01	1.40e−02
cD4_9	[-0.96, 0.88]	[-1.06, 0.81]	[-1.99, 2.04]	3.69e−01	6.57e−01	3.30e−01
cD4_10	[-0.82, 0.87]	[-0.39, 1.04]	[-2.17, 2.06]	1.46e−04	6.14e−01	1.63e−02
cD4_11	[-0.78, 0.89]	[-0.48, 1.23]	[-2.19, 1.47]	1.81e−04	3.85e−03	4.43e−07
cD4_12	[-0.74, 0.73]	[-0.44, 0.93]	[-2.39, 2.49]	5.38e−04	7.52e−01	2.85e−01
cD4_13	[-0.52, 0.49]	[-3.02, 1.43]	[-2.06, 1.36]	1.97e−06	7.83e−03	2.64e−02
cD4_14	[-0.51, 0.51]	[-3.52, 4.09]	[-1.17, 0.95]	2.96e−01	1.87e−01	1.60e−01
cD5_0	[-0.7, 0.73]	[-0.74, 1.76]	[-2.21, 2.32]	1.38e−06	8.10e−01	1.25e−02
cD5_1	[-0.91, 0.93]	[-0.71, 0.96]	[-2.25, 0.84]	1.93e−01	4.01e−08	9.97e−11
cD5_2	[-1.0, 0.96]	[-0.42, 1.38]	[-1.37, 2.27]	2.61e−07	1.58e−03	8.47e−01
cD5_3	[-0.83, 0.56]	[-1.12, 0.1]	[-1.3, 3.72]	1.93e−08	1.54e−12	3.84e−19
cD5_4	[-0.78, 0.81]	[-1.16, 0.63]	[-2.02, 2.37]	1.01e−03	3.26e−01	8.50e−03
cD5_5	[-0.74, 0.85]	[-0.37, 1.29]	[-2.82, 2.53]	7.77e−07	3.18e−01	2.39e−03
cD5_6	[-1.03, 0.98]	[-1.23, 0.94]	[-2.46, 2.23]	2.64e−01	6.34e−01	8.66e−01
cD6_0	[-0.7, 0.56]	[-0.45, 1.95]	[-2.33, 2.91]	2.59e−16	5.87e−02	2.53e−02
cD6_1	[-1.0, 0.86]	[-1.36, 0.75]	[-2.11, 1.5]	1.92e−02	1.01e−01	9.86e−01
cD6_2	[-0.84, 0.83]	[-0.5, 0.91]	[-1.8, 2.12]	6.28e−03	2.59e−01	7.75e−01
cD6_3	[-0.75, 0.73]	[-2.65, 1.1]	[-2.01, 1.77]	1.23e−07	4.17e−01	6.06e−04
cD7_0	[-0.73, 0.85]	[-0.22, 1.43]	[-2.88, 1.84]	6.75e−11	9.81e−04	4.91e−10
cD7_1	[-0.85, 0.88]	[-0.94, 1.11]	[-2.46, 2.09]	4.67e−01	2.36e−01	1.22e−01

The *RR*-interval is the time distance between two successive *R* peaks. Specifically, the interval between the current *R* peak and the previous *R* peak is known as *pre-RR*, while the interval between current *R* peak and the following *R* peak is *post-RR*. The *RR*-interval is one of the most indispensable features used for heartbeat classification. Zhancheng et al. [2] have done extensive work to prove that *pre-RR* is the top distinguishing feature for recognizing *S* beats. Table 1 shows the p-value of *pre-RR* between class *N* and *S* is 2.16e−58, which means that *pre-RR* leads to a significant difference between the *N* and *S* beats.

The *skewness* (3rd order statistics) and the *kurtosis* (4th order statistics) are effective in estimating shape parameters of ECG signals. They are able to well distinguish *V* beats because the major difference of *V* beats against other types of heartbeats is the shape. The corresponding p-values in Table 1 justify this statement.

The *wavelet coefficients* provide multi-frequency-bands information of signals. Since each heartbeat only contains 235 data samples, the maximum level of wavelet decomposition is up to 7. As reported by Asl et al. [12], each type of heartbeats can find its own representative and distinct components in the detail information at level 4-7. In this study, the detail information at these levels are used to represent morphology-related features of a ECG signal.

In conclusion, each of the above-mentioned features is sensitive to at least one certain type of heartbeats distinct from the others. However, if grouping all these features to form a single feature set to classify all types heartbeats together, it is likely to lead to a poor classification performance. Therefore, a pyramid-like model is proposed to select and organize these features to improve performance.

### Pyramid-like classification model

**Fig 4:**
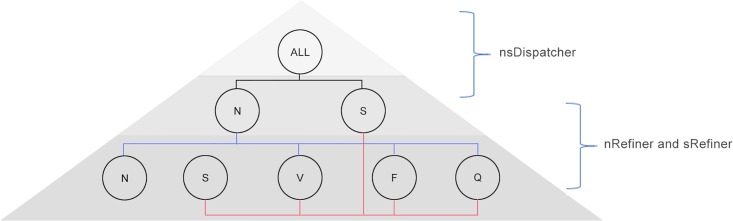
Overall structure of the proposed pyramid-like model.

The proposed pyramid-like model is made up of the *nsDispatcher*, *nRefiner* and *sRefiner*. Fig 4 present the entire framework. The classification process has two stages, known as *level-1* and *level-2* classification. In *level-1* classification, the raw heartbeat data is processed by the *nsDispatcher* at first, where each heartbeat is categorized into the *N* or *S* group. After that, in the *level-2* classification, the *nRefiner* classifies the heartbeats in the upper *N* group to the *N*, *V*, *F* or *Q* group. Simultaneously, the *sRefiner* classifies the heartbeats in the upper *S* group to the *S*, *V*, *F* or *Q* group.

When the shape-related features are included in consideration, *N* and *S* beats are difficult to distinguish, because the *N* and *S* beats share a similar *QRS* complex. Therefore, we focus on classification of *N* and *S* beats specially. In *nsDispatcher*, only the heart rhythm information (*RR*-interval) is considered.

#### Model training

Algorithm 1 presents the training process of *nsDispatcher*. The input training database is denoted as *DS*_*training*_, where each ECG recording represents a patient.

**Algorithm 1:** nsDispatcher Training

**Input:**

A training ECG recordings database, *DS*_*training*_;

**Output:**

Threshold values for each patient, *trsValues*;

1 *step* ← 0.05;

2 **for** *patient in DS*_*training*_ **do**

3  *heartbeats* ← Nomalize(*patient*.*heartbeats*);

4  *pid* ← *patient*.*id*;

5  **for** *hb in heartbeats* **do**

6   **if** *hb.label* ∈ *N* **then**

7    *labelTrue*[*pid*].append(*hb*.*label*);

8    *normalBeats*[*pid*].append(*hb*.*preRR*);

9   **end**

10   **else if** *hb.label* ∈ *S* **then**

11    *labelTrue*[*pid*].append(*hb*.*label*);

12   **end**

13   **else**

14    continue;

15   **end**

16  **end**

17  *normalPreRR* ← median(*normalBeats*[*pid*]);

18  *t* ← 0;

19  **while** *t* > −1 **do**

20   **for** *hb in heartbeats* **do**

21    **if** *hb*.*label* ∉ (*N* ∪ *S*) **then**

22     continue;

23    **end**

24    **else if** *(hb.preRR—hb.postRR) / normalPreRR < t* **then**

25     *labelPred*[*pid*].append(‘S’);

26    **end**

27    **else if** *(hb.preRR—normalPreRR / normalPreRR < t* **then**

28     *labelPred*[*pid*].append(‘S’);

29    **end**

30    **else**

31     *labelPred*[*pid*].append(‘N’);

32    **end**

33   **end**

34   *N*_*Sen*[*t*] ← getSensitivity(′N′, *labelTrue*[*pid*], *labelPred*[*pid*]);

35   *S*_*Sen*[*t*] ← getSensitivity(′S′, *labelTrue*[*pid*], *labelPred*[*pid*]);

36   *t* ← *t* − *step*;

37   *labelPred*[*pid*] ← NULL;

38  **end**

39  *trsValues*[*pid*] ← arg max_*t*_(*N*_*Sen*[*t*] + *S*_*Sen*[*t*]);

40 **end**

The core of the *nsDispatcher* is the decision rules shown between line 20—33 in Algorithm 1. They determine which group (*N* or *S*) a heartbeat belongs to. Let *hb* denote a heartbeat and *t* be the threshold value, the decision rules can then be mathematically expressed as

**rule 1**:

$$\begin{array}{c} \frac{hb.preRR-hb.postRR}{normalRreRR}<t, \end{array}$$ (14)

and **rule 2**:

$$\begin{array}{c} \frac{hb.preRR-normalRreRR}{normalRreRR}<t, \end{array}$$ (15)

where *normalRreRR* represents the median value of the *pre-RR* values of the normal heartbeats.

The rules are motivated by two observations: (1) a *S* beat generally has a shorter *pre-RR* value than that of a surrounding *N* beat; and (2) the gap of the *pre-RR* value between a *S* beat and a *N* beat varies with patients. Therefore, a heartbeat should not be treated as an independent data sample, but be associated with the surrounding beats as well as the patient-specific information. The **rule 1** uses the surrounding beats to help classification. Suppose that in an ECG recording, there is a *S* beat followed by a *N* beat. The *S* beat can be easily caught by the **rule 1**. However, when there are two successive *S* or *N* beats, the **rule 1** can fail because there is not enough information. As such, the **rule 2** is applied to complement the **rule 1** by taking advantage of the patient-specific information (*normalPreRR*).

If any of the rules is satisfied, the heartbeat is categorized as class *S*, otherwise as class *N*. The goal of the training process is to find out the best threshold value (*t*) that helps to achieve a high detection sensitivity of both the *N* and *S* beats for the decision rules of each patient. We traverse every possible *t* in the range of (−1, 0). Values beyond this range is practically impossible so far. The parameter *step* is used to control the precision of *t*. The smaller the *step*, the more precise the *t* but the more time-consuming the training process. Formally, the objective function (line 39 in Algorithm 1) is formulated as:

$$\begin{array}{c} \underset{t}{\arg\max}\left(N\_Sen\left[t\right]+S\_Sen\left[t\right]\right). \end{array}$$ (16)

The trained threshold values are stored in *trsValues* (line 39 in Algorithm 1).

In terms of the *nRefiner* and the *sRefiner*, Table 2 summarize their compositions and the training features. Notice that the *N* group is seriously imbalanced and dominated by the normal heartbeats. To reduce the impact caused by the imbalance problem, a mix classifier ensemble method is applied in the *nRefiner*. The reason for excluding the heartbeat rhythm for training the *sRefiner* is that the *V* beats could also have irregular *RR*-interval values as the *S* beats.

**Table 2:** The nRefiner and the sRefiner.

	Classifier	Features
nRefiner	Mix Ensemble(Linear SVM, SVM, Decision Tree, KNN, Logistic Regression, Perceptron, and Bayes)	heartbeat rhythm, HOS, and wavelet coefficients
sRefiner	SVM	HOS and wavelet coefficients

#### Classification

The details of *level-1* and *level-2* classification are presented in Algorithm 2 and Algorithm 4, respectively.

In *level-1* classification, one important step is the estimation of the normal *pre-RR* value of a patient (line 4—11 in Algorithm 2). For each patient *p*_*a*_ in *DS*_*test*_, we perform a statistical analysis on *p*_*a*_’s heartbeat *pre-RR* values via *boxploting*. If less than 10% of the data are considered as outliers, we assume that the ECG recording is dominated by the normal heartbeats and use

$$\begin{array}{c} E(normalRreRR)\leftarrow median(heartbeats.preRRs) \end{array}$$ (17)

to estimate the normal *pre-RR* value. Such an assumption is practical and reasonable because *S* beats occur sparsely in real-world applications. On the other hand, if more than 10% of the data are considered as outliers, the ECG recording is likely to be distorted by the *S* beats and *median*(*heartbeats*.*preRRs*) could represent the *pre-RR* value of a *S* beat. In such a case, we use

$$\begin{array}{c} E(normalPreRR)\leftarrow mean(mean(outliers),median(heartbeats.preRRs)) \end{array}$$ (18)

to estimate the normal *pre-RR* value. This guarantees that the *E*(*normalPreRR*) is not representing an irregular value.

**Algorithm 2:** Level-1 Classification

**Input:**

A test ECG recordigns database, *DS*_*test*_;

The trained threshold values, *trsValues*;

**Output:**

The result of level-1 classification, *lev*1*Result*

1 **for** *patient in DS*_*test*_ **do**

2  *pid* ← *patient*.*id*;

3  *heartbeats* ← Nomalize(*patient*.*heartbeats*);

4  *stats* ← boxplot(*heartbeats*.*preRRs*);

5  *outliers* ← *stats*.*outliers*;

6  **if** *len(outliers*) / *len(heartbeats*) >0.1 **then**

7   *E*(*normalPreRR*) ← mean(mean(*outliers*),

    median(*heartbeats*.*preRRs*));

8  **end**

9  **else**

10   *E*(*normalPreRR*) ← median(*heartbeats*.*preRRs*);

11  **end**

12  *neighbor* ← getNeighbor(*patient*);

13  *t* ← *trsValues*[*neighbor*];

14  **if** *t* equals to 0 **then**

15   *t* ← min(trsValues)

16  **end**

17  **for** *hb in heartbeats* **do**

18   **if** *(hb.preRR—hb.postRR)* / *E(normalPreRR)* < *t* **then**

19    *lev*1*Result*[*pid*].append(‘S’);

20   **end**

21   **else if** *(heartbeat.preRR—E(normalPreRR))* / *E(normalPreRR)* < *t*

    **then**

22     *lev*1*Result*[*pid*].append(‘S’);

23   **end**

24   **else**

25     *lev*1*Result*[*pid*].append(‘N’);

26   **end**

27  **end**

28 **end**

The algorithm goes on by looking for a patient *p*_*b*_ in *DS*_*training*_ who has the most similar *pre-RR* values distribution with *p*_*a*_, and assign *p*_*b*_’s threshold value to *p*_*a*_ (line 12—13 in Algorithm 2). We implement a function named *getNeighbor* (Algorithm 3) to perform the task. The function uses the *Earth mover’s distance* (EMD) to measure the dissimilarity of two distributions. Notice that if *p*_*b*_’s threshold value equals to 0, which means that no *S* beat is found in *p*_*b*_, it is believed that there is also a low probability to find *S* beats in *p*_*a*_. However, we never want to miss a potential *S* beat, which may lead to a serious consequence to a patient. In such a case, we assign the smallest value in *trsValues* to *p*_*a*_ (line 14—16 in Algorithm 2). This implies that the algorithm try to search for the potential *S* beats while avoid classifying the *N* beats as *S* beats.

Once the *E*(*normalPreRR*) as well as the *t* are ready, the heartbeats are processed by the decision rules.

**Algorithm 3:** find the nearest neighbor of a patient

**Input:**

An ECG recording of a patient, *testPatient*;

The training ECG recordings database, *DS*_*training*_;

**Output:**

A patient in *DS*_*training*_ who has the most similar *previour-RR* values

distribution of the *testPatient*, *neighbor*

1 **Function** *getNeighbor(testPatient)* **is**

2  *data*1 ← Normalize(*testPatient*.*heartbeats*.*preRRs*);

3  **for** *trainPatient in DS*_*training*_ **do**

4   *pid* ← *trainPatient*.*id*;

5   *data*2[*pid*] ← Normalize(*trainPatient*.*heartbeats*.*preRRs*);

6  **end**

7  *neighbor* ← arg max_*pid*_(EMD(*data*1, *data*2[*pid*]));

8  return *neighbor*;

9 **end**

In *level-2* classification (Algorithm 4), each heartbeat in the *N* group is further classified by the *nRefiner* to class *N*, *V*, *F* or *Q*. Similarly, the *sRefiner* reclassified the *S* beats to class *S*, *V*, *F* or *Q*.

**Algorithm 4:** Level-2 Classification

**Input:**

The test ECG recordings database, *DS*_*test*_;

The level-1 classification result, *lev*1*Result*;

**Output:**

The final result of the pyramid-like model, *finalResult*;

1 **for** patient in *DS*_*test*_ **do**

2  *pid* ← *patient*.*id*;

3  *heartbeats* ← Nomalize(*patient*.*heartbeats*);

4  **for** *hb in heartbeats* **do**

5   **if** *lev1Result[pid][hb.id]* ∈ *N* **then**

6    *finalResult*[*pid*].append(nRefiner(*hb*));

7   **end**

8   **else if** *lev1Result[pid][hb.id]* ∈ *S* **then**

9    *finalResult*[*pid*].append(sRefiner(*hb*));

10   **end**

11   **else**

12    continue;

13   **end**

14  **end**

15 **end**

## Experimental ECG databases

In this section, three ECG databases are introduced, namely the *MIT-BIH-AR* database and the *INCART* database. They are public-accessible from the *Physiobank* [33]. S1 File contains hyper links for downloading the data.

Most of the works on heartbeat classification trained and evaluated their models on the *MIT-BIH-AR* database. In order to have a fair comparison, both the training and the evaluation of the pyramid-model is performed on the *MIT-BIH-AR* database as well. Besides, we use the *INCART* database to assess the generalization performance of the proposed model.

All ECG recordings in these databases have an equal length of 30 minutes, but they are not sampled in the same frequency. They need to be re-sampled to 360*Hz* before use. The recordings are well-labeled in heartbeat level. The original heartbeat annotations include 15 classes, which are further grouped into 5 super-classes by the *AAMI* [1], as shown in Table 3.

**Table 3:** ECG-based heartbeat annotations.

AAMI class	Original class	Type of beat
Normal (*N*)	*N*	Normal beat
	*L*	Left bundle branch block beat
	*R*	Right bundle branch block beat
	*e*	Atrial escape beat
	*j*	Nodal (junctional) escape beat
Supraventricular ectopic beat (*S*)	*A*	Atrial premature beat
	*a*	Aberrated atrial premature beat
	*J*	Nodal (junctional) premature beat
	*S*	Supraventricular premature beat
Ventricular ectopic beat (*V*)	*V*	premature ventricular contraction
	*E*	Ventricular escape beat
Fusion beat (*F*)	*F*	Fusion of ventricular and normal beat
Unknown beat (*Q*)	/	Paced beat
	*f*	Fusion of paced and normal beat
	*Q*	Unclassifiable beat

Details of these databases are respectively given below.

### MIT-BIH-AR database

The database contains 48 two-lead ambulatory ECG recordings from 47 patients (including 22 females and 25 males). Each recording is denoted by a 3-digits number. The recordings were digitized at 360*Hz* per second per channel with 11-bit resolution over a 10 − *mV* range. For most of them, the first lead is modified limb lead II (except for the recording 114). The second lead is a pericardial lead (usually *V1*, sometimes are *V2*, *V4* or *V5*, depending on subjects). In this study, only the modified limb lead II is used.

The database is seriously imbalanced. The *N* beats dominate most of the recordings. Therefore, the *k*-fold validation scheme cannot be applied to split the database for training and testing. Two different paradigms are found in the literature to solve this problem [2, 3, 6, 7]. One is the intra-patient paradigm, which first mixes up the heartbeats from all recordings and then evenly allocates each category of heartbeats into two groups. The other one is the inter-patient paradigm. In this paradigm, the ECG recordings are divided into two datasets (*DS*1 and *DS*2) with each dataset containing approximately the same portion of heartbeat classes. Table 4 shows the division and the corresponding heartbeat classes distribution. The *DS*1 is used for model training and the *DS*2 is used for model performance evaluation.

**Table 4:** The inter-patient division paradigm.

Data set	N	S	V	F	Q	Recordings^1^^,^^2^
DS1	45808	943	3786	414	8	101, 106, 108, 109, 112, 114, 115, 116, 118, 119, 122, 124, 201, 203, 205, 207, 208, 209, 215, 220, 223, 230
DS2	44198	1836	3219	388	7	100, 103, 105, 111, 113, 117, 121, 123, 200, 202, 210, 212, 213, 214, 219, 221, 222, 228, 231, 232, 233, 234

^1^ Each recording is denoted by a 3-digits number and the numbers are originally discontinuous.

^2^ As recommended by the AAMI, the four recordings (102, 104, 107 and 217) containing paced beats are excluded from the analysis.

It has been empirically proven that the intra-patient paradigm can bias the classification result by allowing training and testing heartbeats coming from the same patient [9]. By contrast, the inter-patient paradigm is more objective. In order to reveal the true performance of the pyramid-like model and have a fair comparison with the stat-of-the-art rivals, the inter-patient paradigm is strictly followed in this work.

### INCART 12-leads arrhythmia database

This database consists of 75 ECG recordings sampled at 257*Hz*. Each recording contains 12 standard leads. Similarly, only the modified limb lead II is used in this study. The annotations were first produced by an automatic algorithm and then corrected manually based on the standard PhysioBank beat annotation definitions. None of the recordings contains pacemakers, but most of them have ventricular ectopic beats. The heartbeat distribution of the *INCART* database is shown in Table 5.

**Table 5:** Heartbeat distributions in the INCART database.

Database	N	S	V	F	Q
INCART	153491	1958	19993	219	6

## Experimental evaluation

In this section, we conduct a benchmark evaluation for the proposed pyramid-like model on the *MIT-BIH-AR* database, with the result being compared to the state-of-the-art methods. Besides, we use the *INCART* database to assess the model generalization performance.

All the experiments presented in this work are programmed in Python 3.63 and done in a 64-bits Windows 10 PC, with *i*5 − 4590 CPU and 12 GB memory.

### Evaluation metrics

In this work, the performance is evaluated by sensitivity (*Se*), positive predictive value (+*P*) and accuracy value (*Acc*) as follows, where *TP*, *TN*, *FP* and *FN* denotes *true positive*, *true negative*, *false positive* and *false negative*, respectively, and ∑ represents the amount of instances in the data set.

$$\begin{array}{c} Se=\frac{TP}{TP+FN} \end{array}$$ (19)

$$\begin{array}{c} +P=\frac{TP}{TP+FP} \end{array}$$ (20)

$$\begin{array}{c} Acc=\frac{TP+TN}{\sum } \end{array}$$ (21)

It should be noted that penalties would not be applied for the misclassification of class *F* and *Q*, as recommended by the *AAMI* standard.

### Classification result and discussion

Table 6 shows the result of the *level-1* classification. The majority of the *N* and *S* beats are correctly classified by the *nsDispatcher*. Although 3153 *N* beats are misclassified as *S* beats, they only account for a small portion of the total *N* beats. A good classification sensitivity and positive predictive value of the *N* beat is still achieved. On the other hand, the misclassified *N* beats lead to a decrease of the positive predictive value of the *S* beats. However, as the heartbeat classification plays an important role toward identifying the cardiac arrhythmia, the accuracy over the class *S* is considered most important [28]. From an overall point of view, the *nsDispatcher* does a decent job.

**Table 6:** The result of level-1 classification of the proposed model on DS2.

		Predicted class	
		N	S
True class	N	40918	3151
	S	74	1680
	V	872	2347
	F	383	5
	Q	5	2

Table 7 gives the final classification results of the proposed pyramid-like model in detail. It is worth noting that, form *level-1* to *level-2* classification, only 164 *N* beats and 87 *S* beats are misclassified by the *nRefiner* and the *sRefiner*. In addition, the *level-2* classification achieves superior performance in detection of the *V* beats. The results indicate the effectiveness of the *nRefiner* and the *sRefiner*. In terms of the *F* and *Q* beats, a poor performance is obtained, which is a normal phenomenon because both *F* and *Q* beats are originally unclassifiable. The same issue is commonly found in all the existing research works.

**Table 7:** The result of level-2 classification of the proposed model on DS2.

		Predicted class				
		N	S	V	F	Q
True class	N	40754	2762	508	45	0
	S	71	1593	87	3	0
	V	125	151	2856	87	0
	F	317	1	62	8	0
	Q	2	0	4	1	0

The proposed model is compared to the state-of-the-art methods over the same test set (*DS*2). Table 8 summarizes the comparative result. The proposed model exhibits higher performance in terms of the positive predictive value of *N* beats and the sensitivity value of the disease heartbeats (*S* and *V*). In addition, it takes the second best place in global accuracy (91.5%) and the sensitivity value of class *N* (99.0%).

**Table 8:** Performance comparison of the proposed model and the state-of-the-art methods on DS2.

Method	Acc(%)	N		S		V	
		Se(%)	+P(%)	Se(%)	+P(%)	Se(%)	+P(%)
**Proposed**	91.5	92.0	**99.0**	**91.0**	35.0	**89.0**	81.0
De Chazal [3]	81.9	86.9	99.2	75.9	38.5	77.7	81.9
Ye C [6]	86.4	88.5	97.5	60.8	**52.3**	81.5	63.1
Zhang Z [2]	86.7	88.9	99.0	79.1	36.0	85.5	**92.8**
Shan C [8]	**93.1**	**98.4**	95.4	29.5	38.4	70.8	85.1
Mariano L [4]	78.0	78.0	99.0	76.0	41.0	83.0	88.0

Although our model has the lowest positive predictive value of the *S* beats, we make a breakthrough in the sensitivity value (91.0%). Actually, as we can see, the positive predictive values of class *S* are commonly low in most of the existing methods. The best one is obtained by Ye C et al. [6], which is just 17% better than ours, but we beat it in the sensitivity value by more than 30%.

### Generalization result and discussion

The classification result on the *INCART* database is summarized in Table 9. The performance is compared to the latest work by Mariano L. and Juan P. [4], which is the only work can be found performing model evaluation on both the *MIT-BIH-AR* and the *INCART* database. Table 10 presents the comparative result.

**Table 9:** Classification result of the proposed pyramid-like model in the INCART database.

		Predicted class		
		N	S	V
True class	N	138620	6871	8000
	S	106	1554	298
	V	792	1643	17783

**Table 10:** Generalization performance comparison between the proposed model and the stat-of-the-art rival in the INCART database.

Method	Acc(%)	N		S		V	
		Se(%)	+P(%)	Se(%)	+P(%)	Se(%)	+P(%)
**Proposed**	90.0	90.3	**99.3**	79.4	**15.4**	**87.0**	72.7
Mariano L [4]	**91.0**	**92.0**	99.0	**85.0**	11.0	82.0	**88.0**

Notice that the compared method [4] follows the *AAMI*2 labeling, where class *F* and *Q* are merged into class *V*. In order to have a fair comparison, we adapt the proposed model to the *AAMI*2 labeling.

As seen from Table 10, the proposed model has a comparable performance with the rival on the *INCART* database. Both the works achieve similar values in all metrics. However, if we look back at Table 8, the proposed pyramid-like model presents better performance on *DS*2.

It is worth noting that, from *DS*2 to the *INCART* database, the proposed model maintains a stable heartbeat classification performance. This is very important, as robustness is indispensable for an algorithm to be applied in a clinical practice.

## Conclusion

Millions of people around the world are suffering from the cardiac arrhythmia. Automatic heartbeat classification helps early identify this issue, making it possible for people to get the right treatment sooner. In this paper, a pyramid-like model has been proposed for automatic heartbeat classification. The model integrates three components, namely *nsDispatcher*, *nRefiner* and *sRefiner*. During the classification process, the *nsDispatcher* first allocates the heartbeats into the *N* or *S* group. The *nRefiner* and the *sRefiner* then further classify the heartbeats in the *N* and *S* group respectively to give the final decision. The significance of the proposed model is that it takes the surrounding heartbeats as well as the patient-specific information into consideration to help identification of a *S* beat. Besides, the *nRefiner* and the *sRefiner* are customized with different classifier structure and training features to adapt to the classification requirements in the *N* and *S* group.

The proposed model has been evaluated on the *MIT-BIH-AR* database, with the performance being compared against the state-of-the-art methods. In addition, the *INCART* database is used to measure the generalization performance of the proposed model. The experimental results have proven the effectiveness and robustness of the proposed model in heartbeat classification.

## Supporting information

S1 File Data and codes. This file contains hypter links for accessing the experiemtal data as well as the codes for the pyramid model.
(PDF)

S2 File The boxplots for all features. This file contains boxplots for all features.
(PDF)
