# Supplementary material for: A pyramid-like model for heartbeat classification from ECG recordings
Source: PLoS One. 2018 Nov 14;13(11):e0206593. doi: 10.1371/journal.pone.0206593 (PMC6235298; doi:10.1371/journal.pone.0206593)
Supplement: S1 File — This file contains hypter links for accessing the experiemtal data as well as the codes for the pyramid model. (PDF) [file pone.0206593.s001.pdf]

Data Availability:

The two ECG databases used in this work can be download from the Physiobank.

MIT-BIH-AR database: https://www.physionet.org/physiobank/database/mitdb/

INCART database: https://www.physionet.org/pn3/incartdb/

Code Availability:

The source codes for this study including data preparation, feature extraction, model training and classification are accessible in https://github.com/SamHO666/A-Pyramid-like-Model-for-Heartbeat-Classification. The instruction for reproducing the experimental results can be found in this Github repository as well.
