# Supplementary figures and images for: A pyramid-like model for heartbeat classification from ECG recordings
Source: PLoS One. 2018 Nov 14;13(11):e0206593. doi: 10.1371/journal.pone.0206593 (PMC6235298; doi:10.1371/journal.pone.0206593)

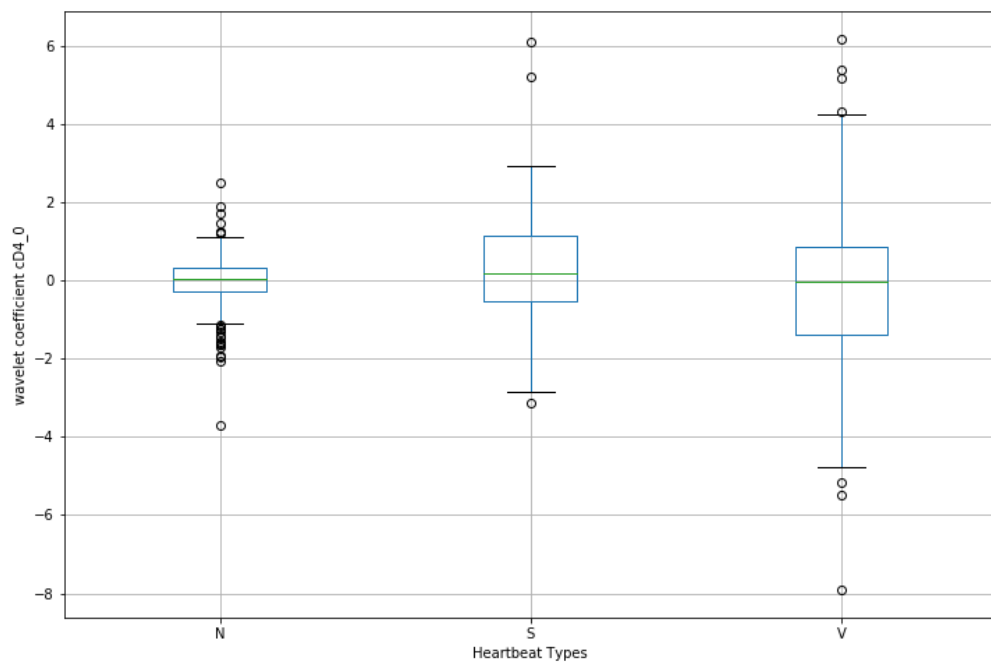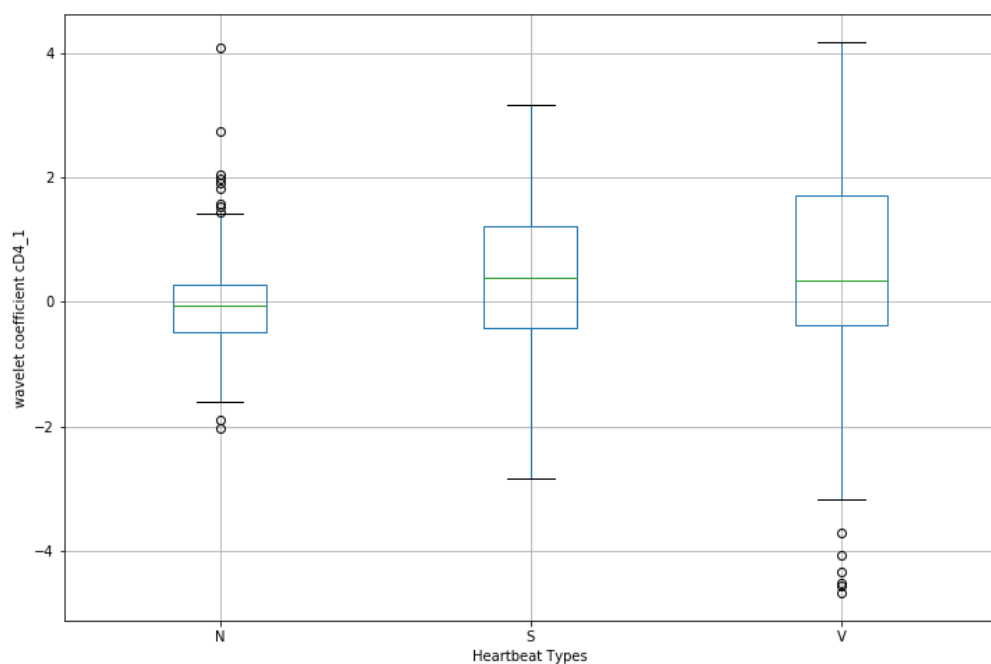

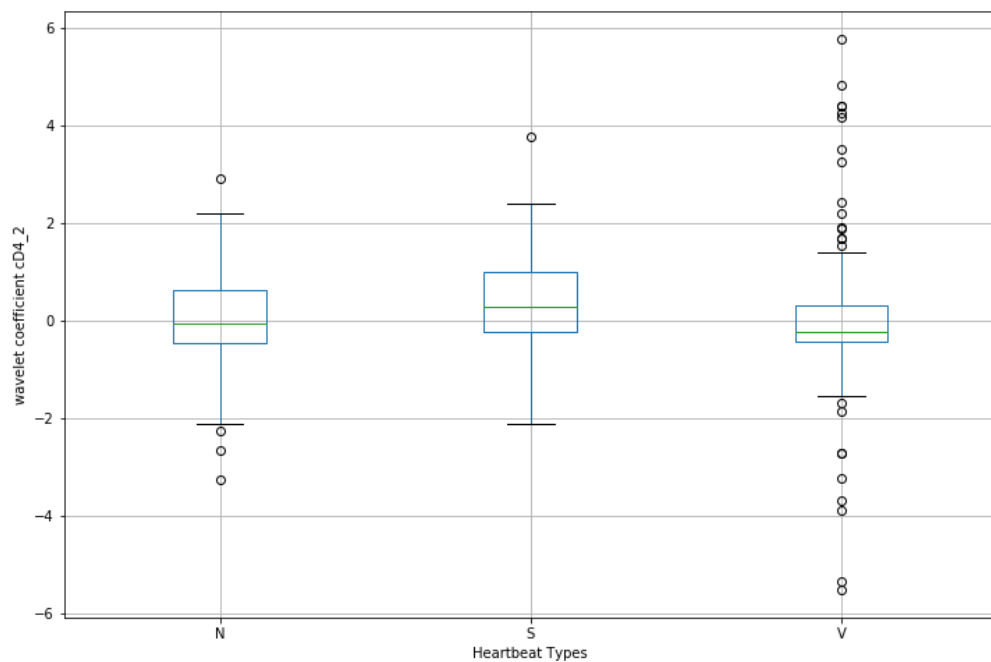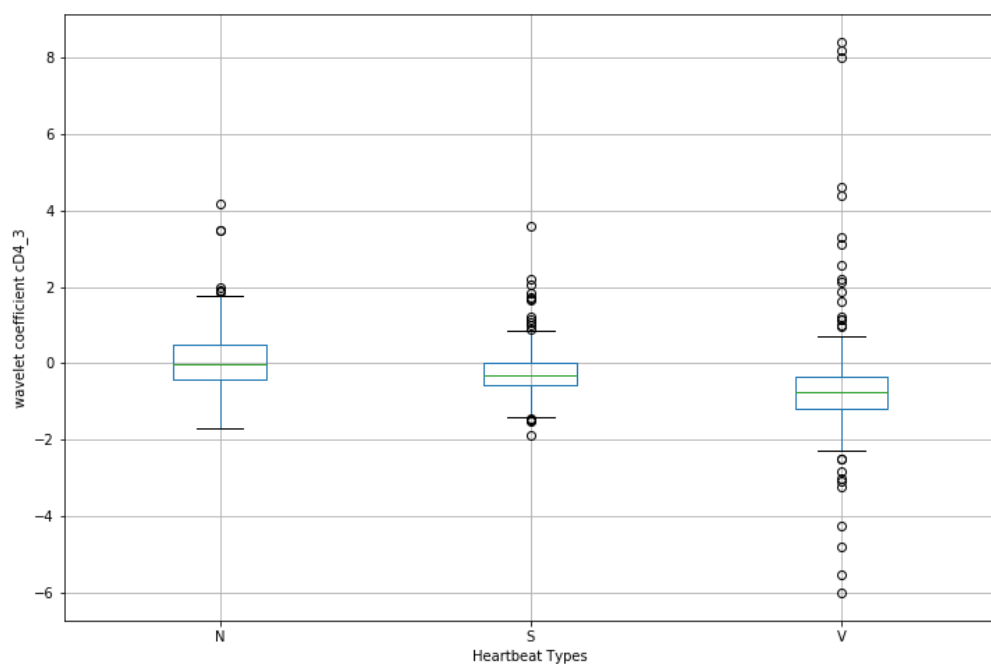

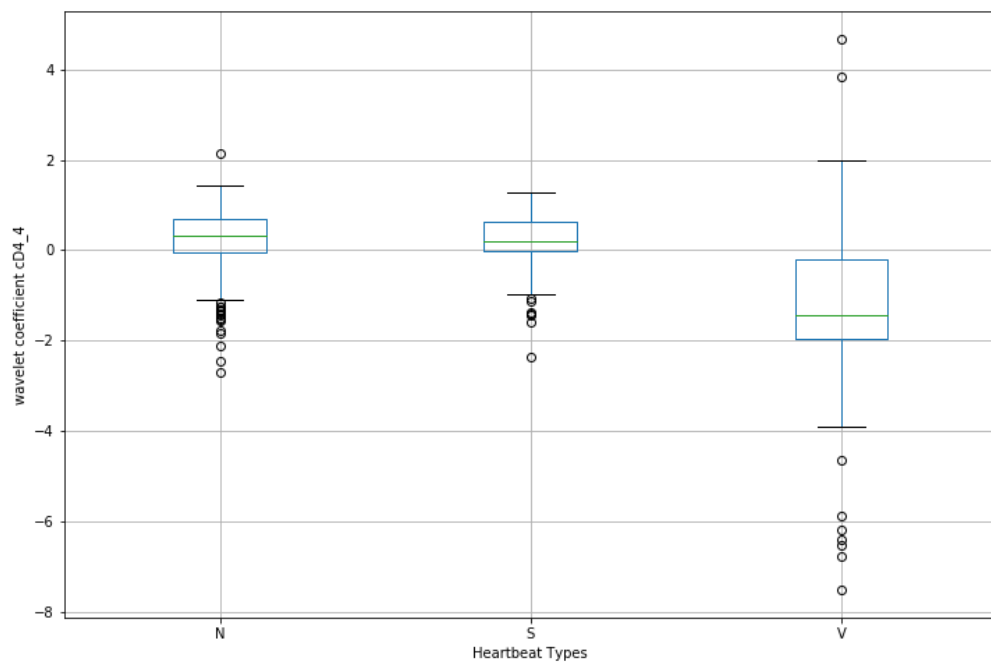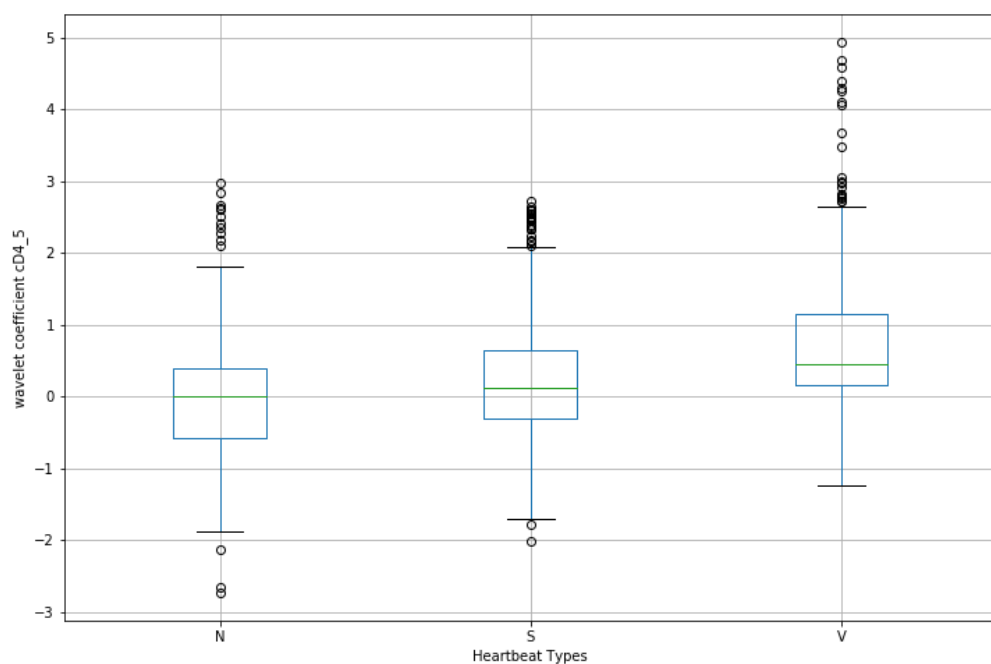

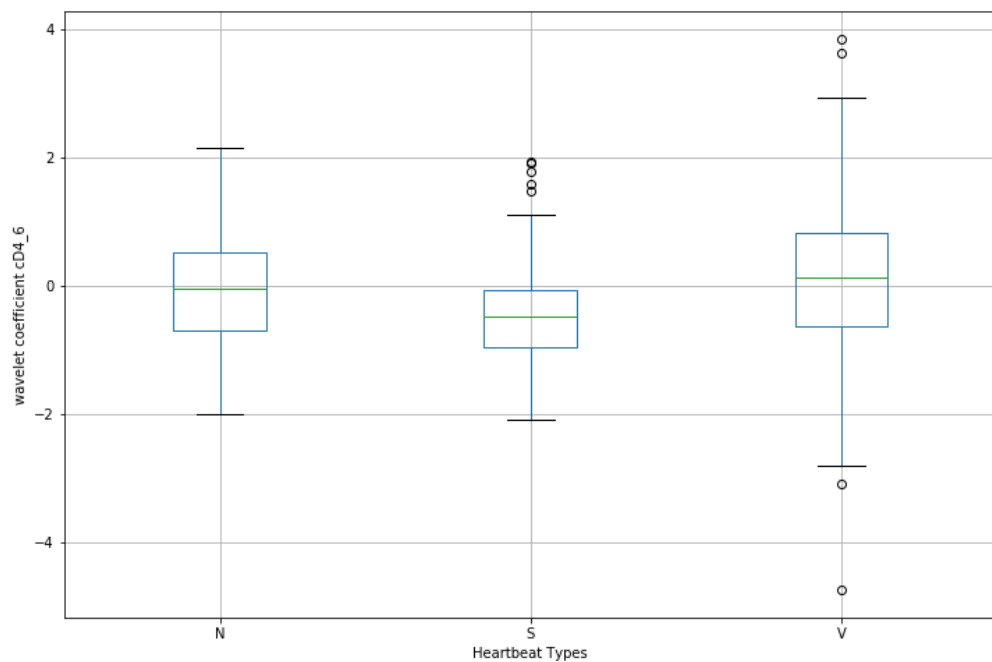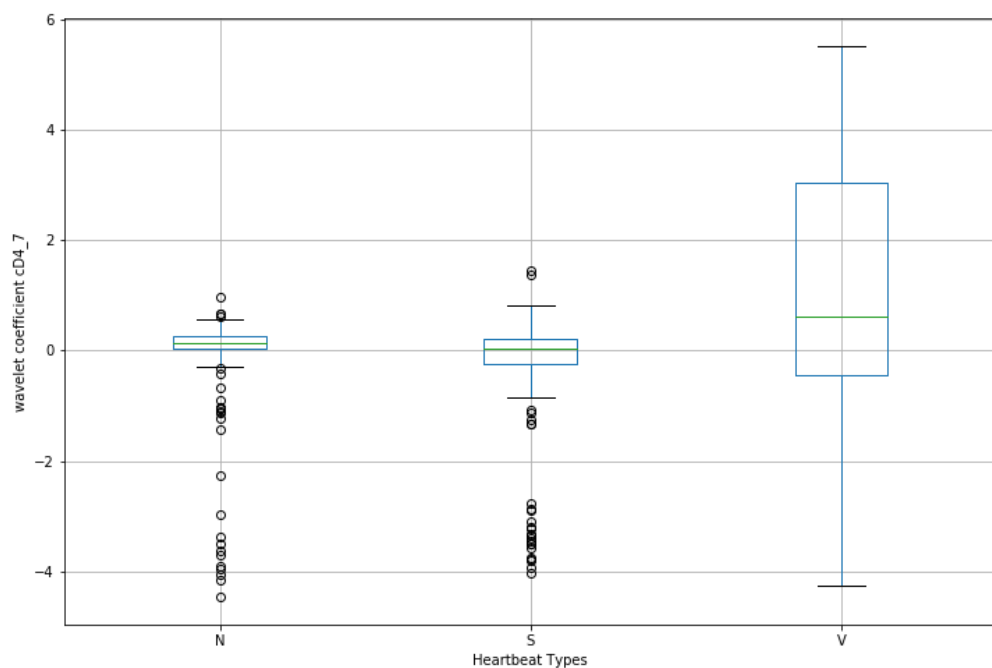

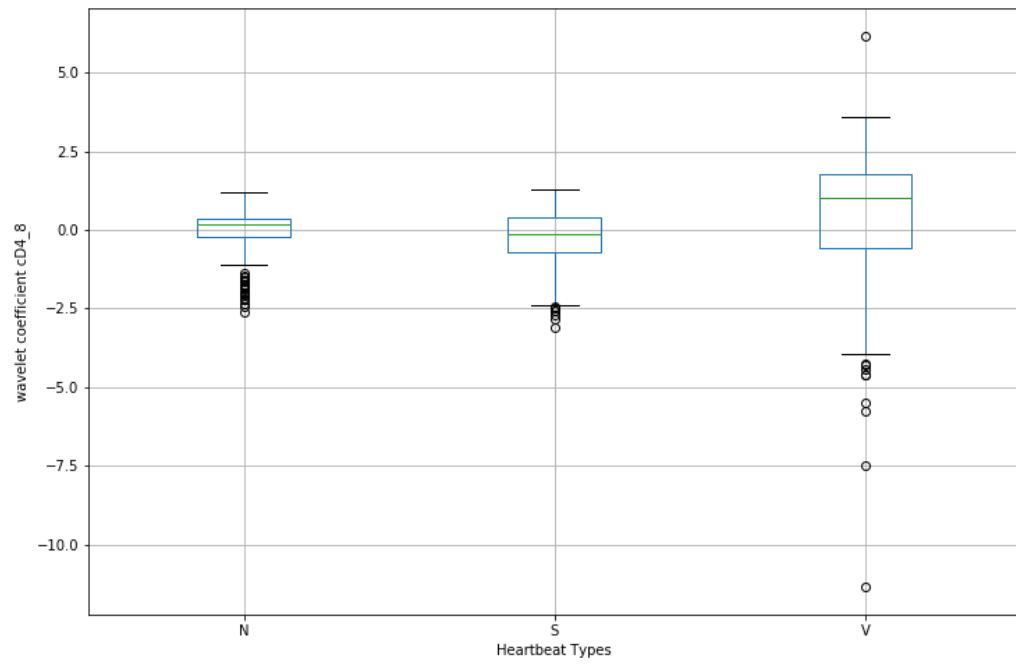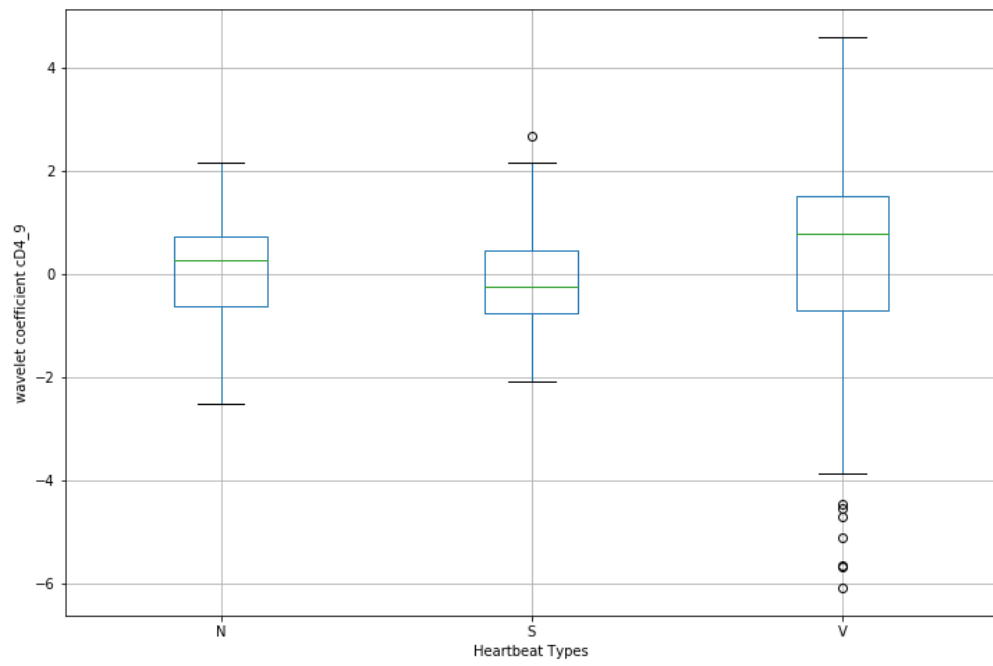

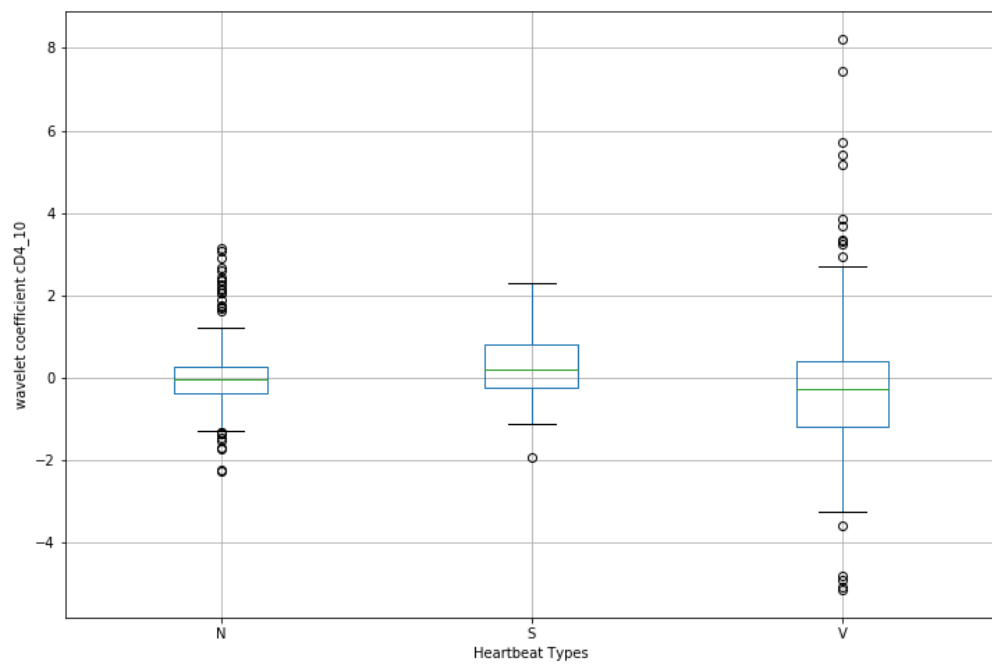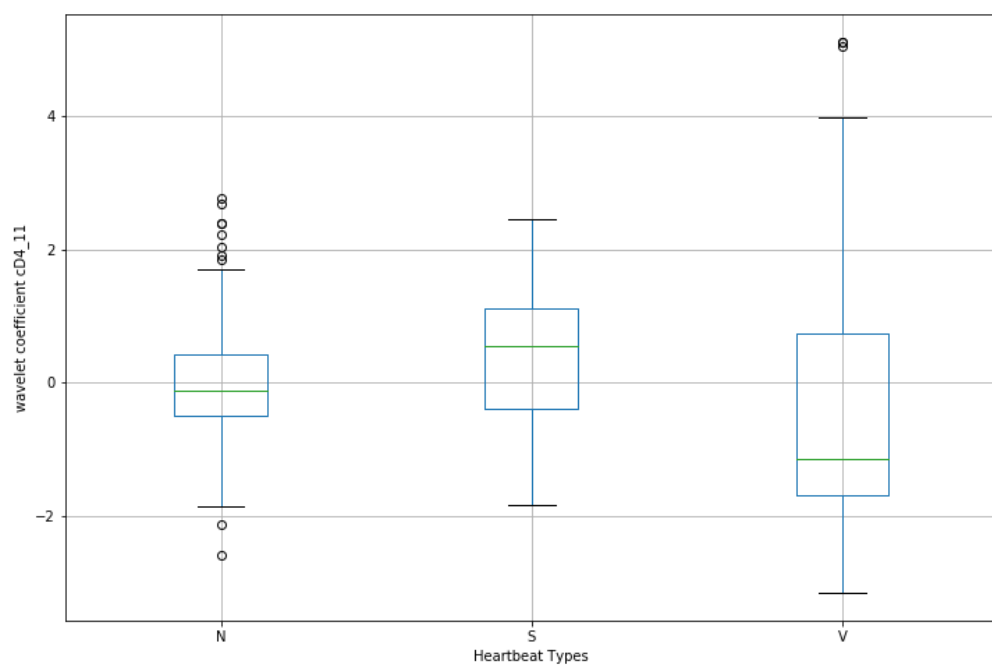

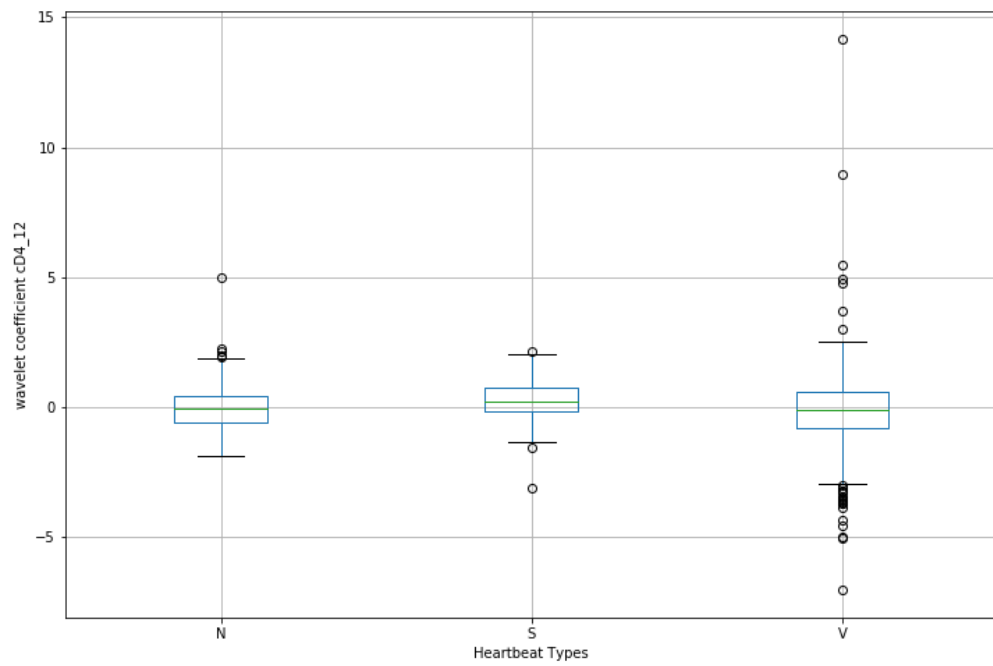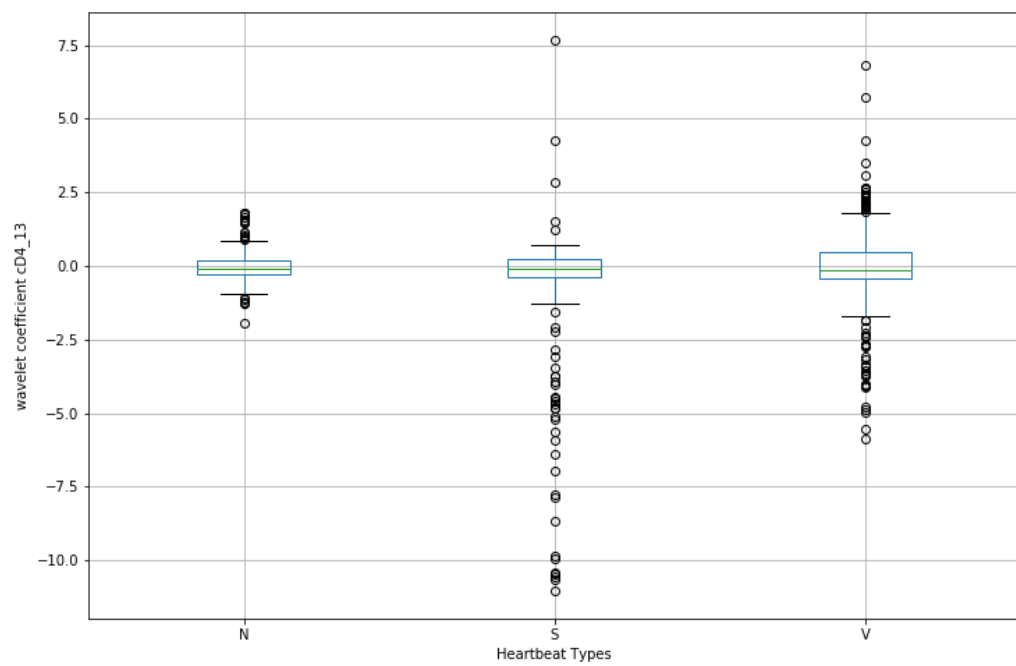

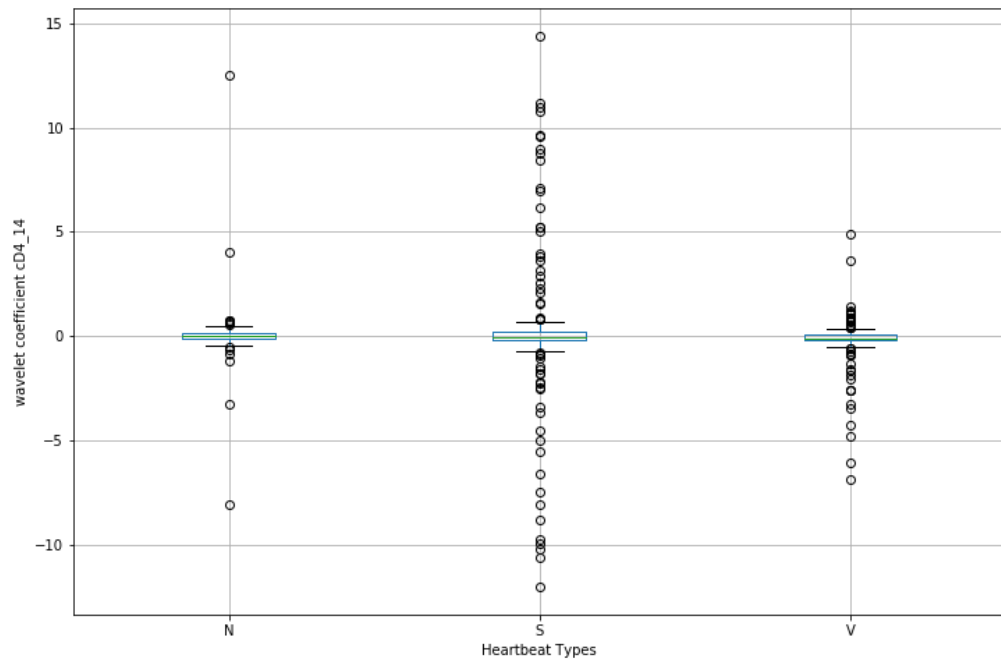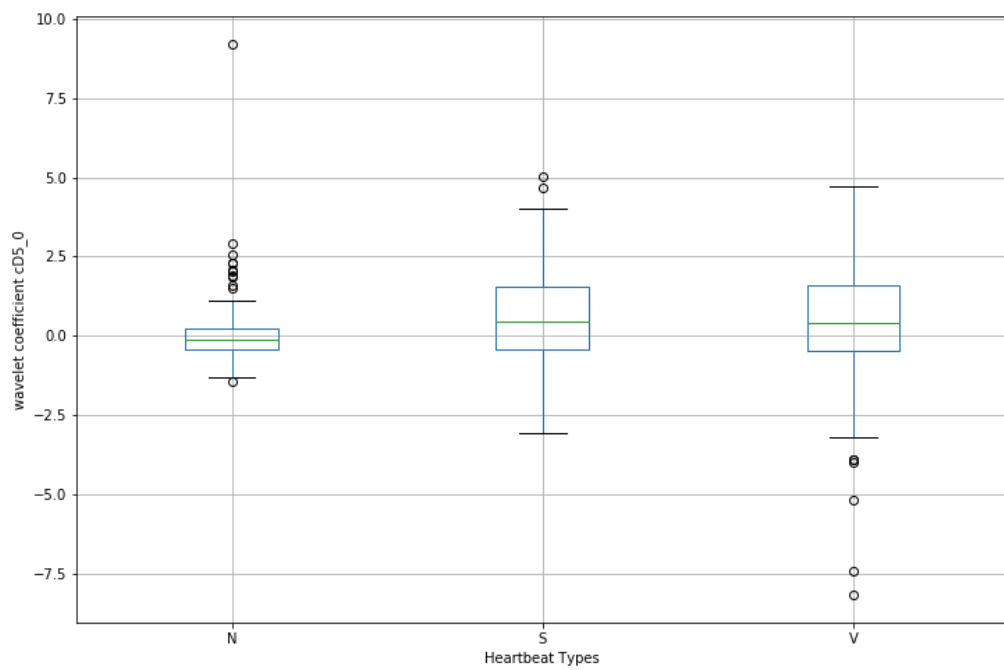

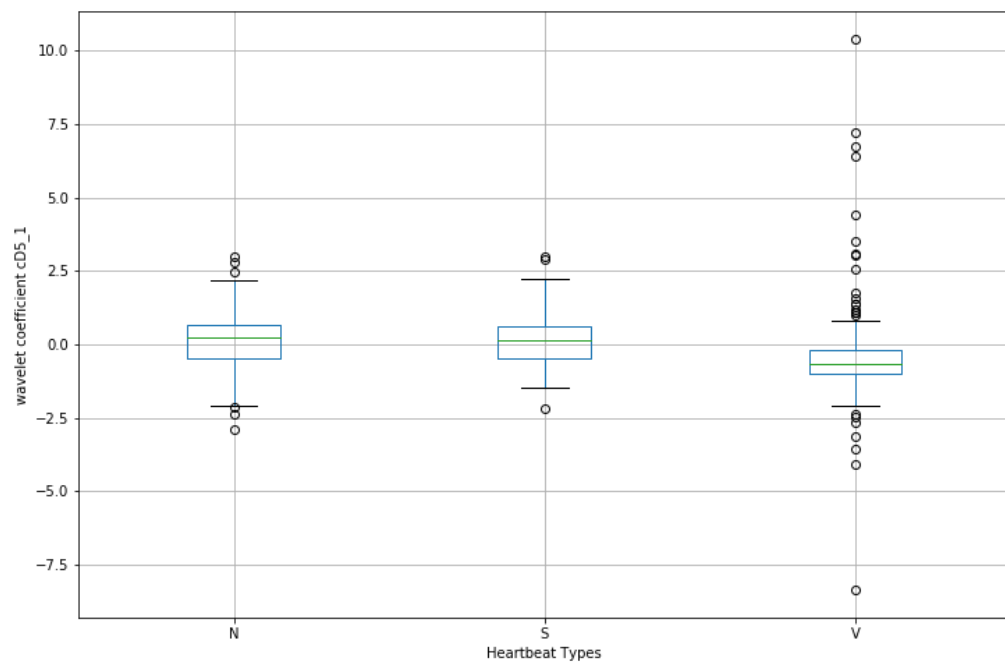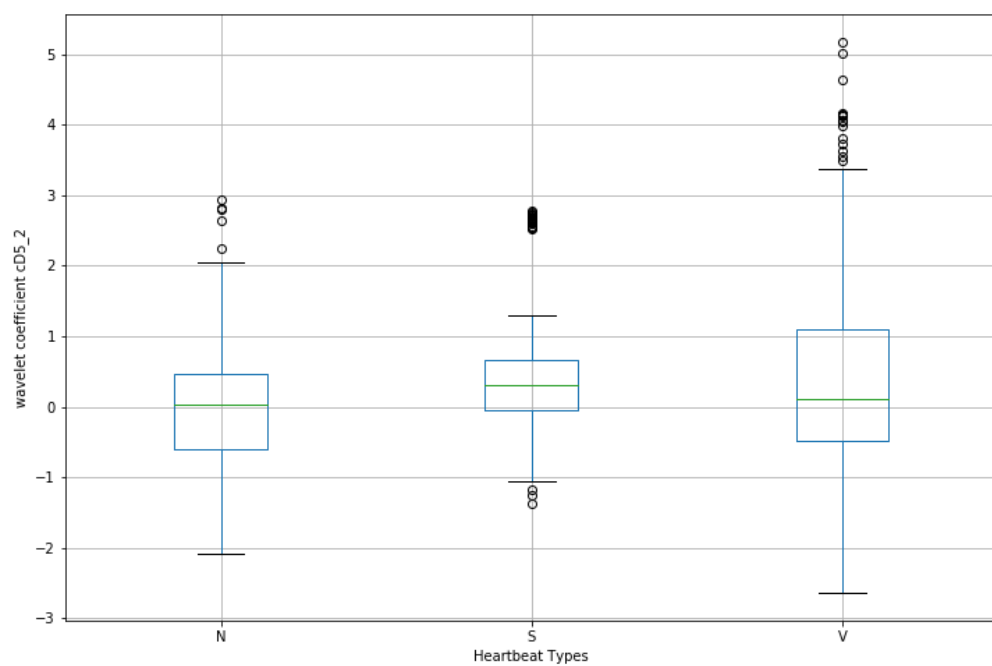

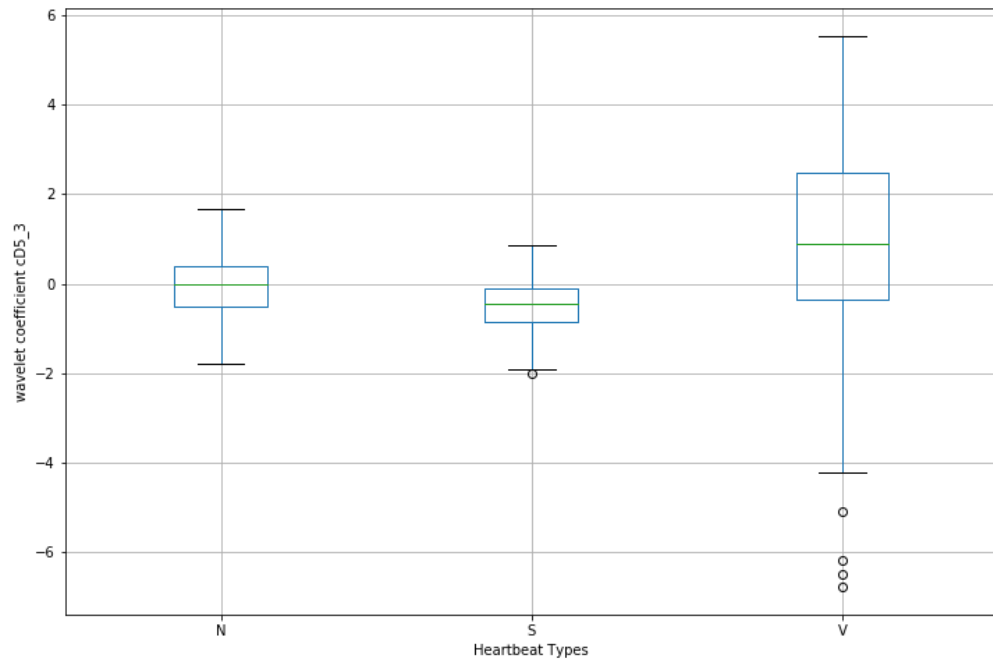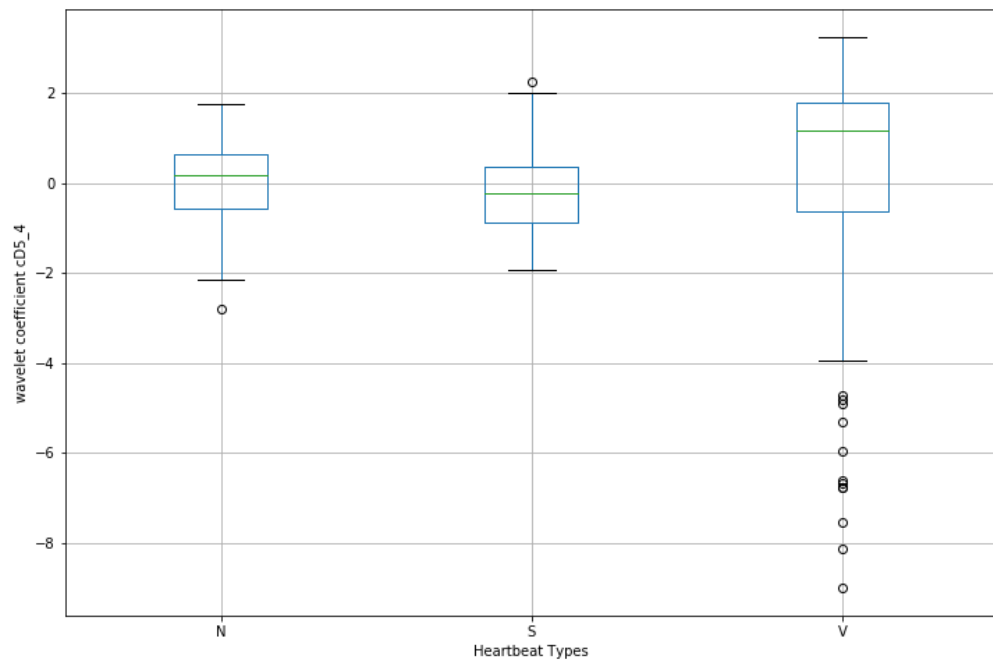

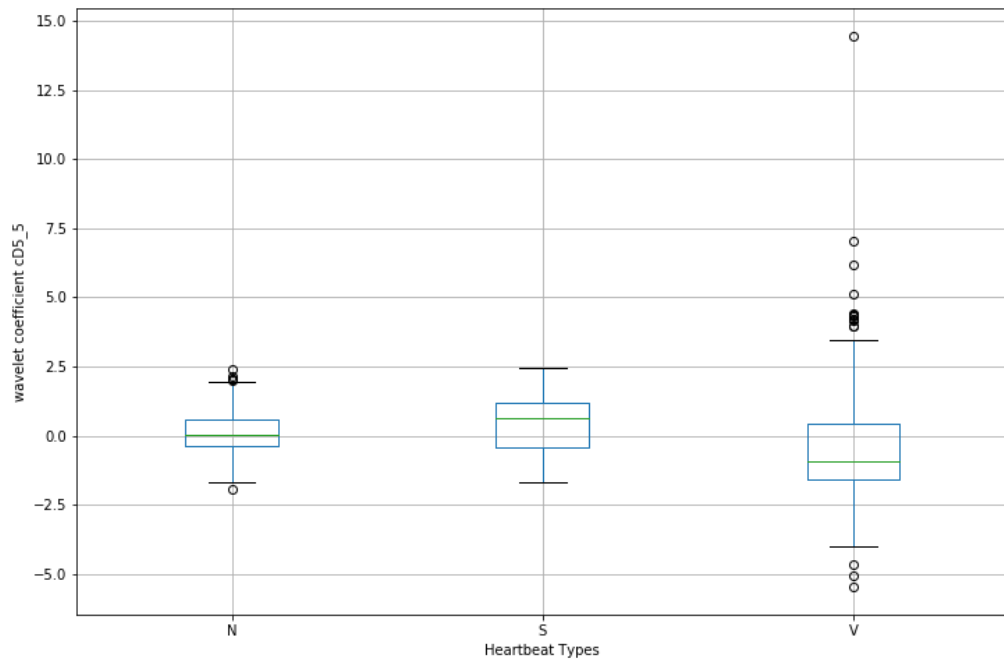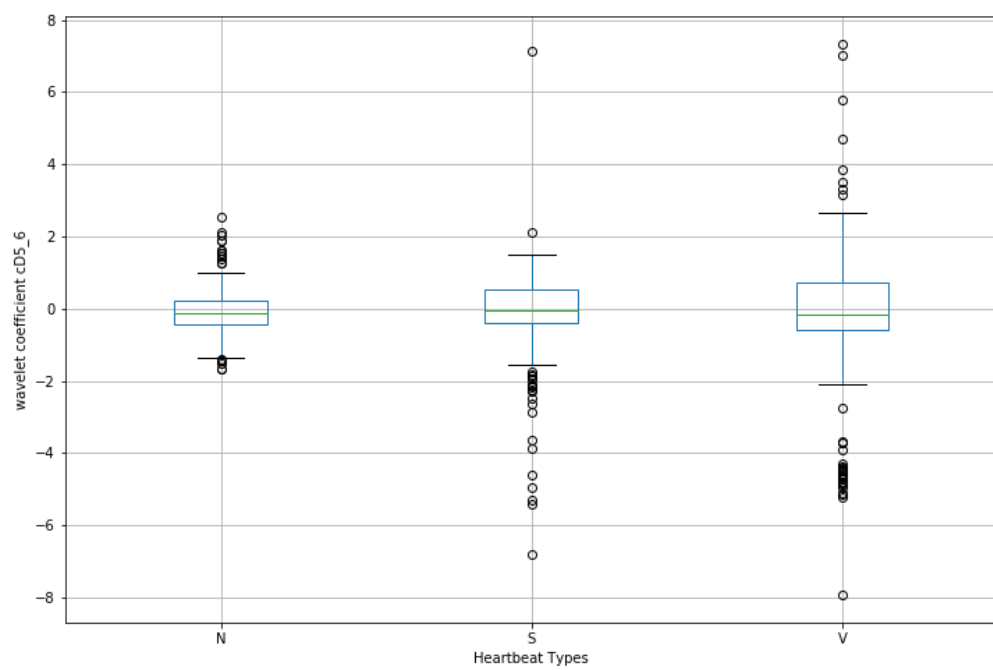

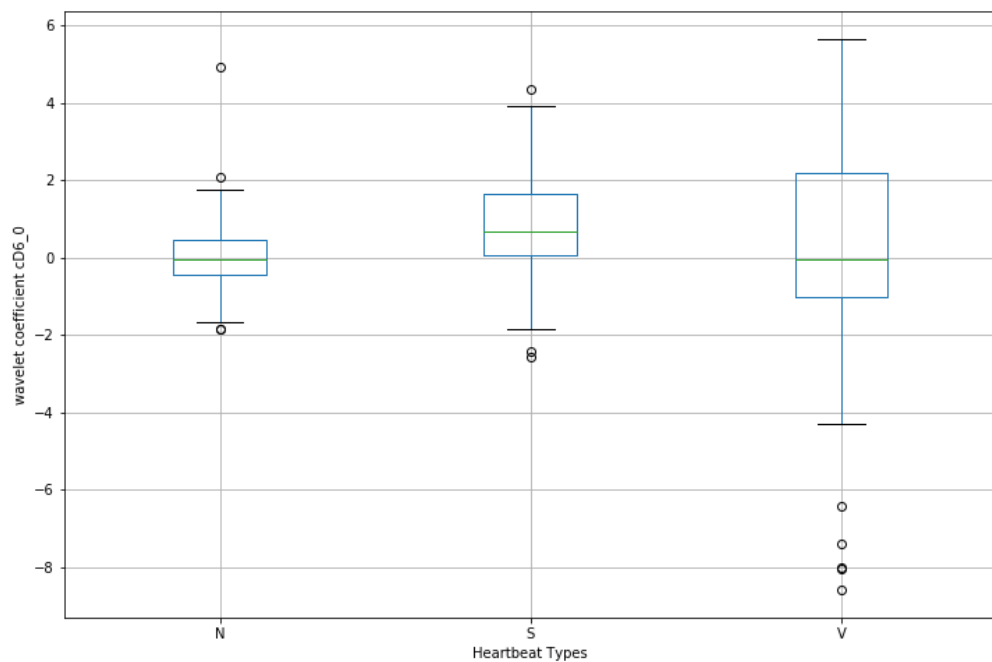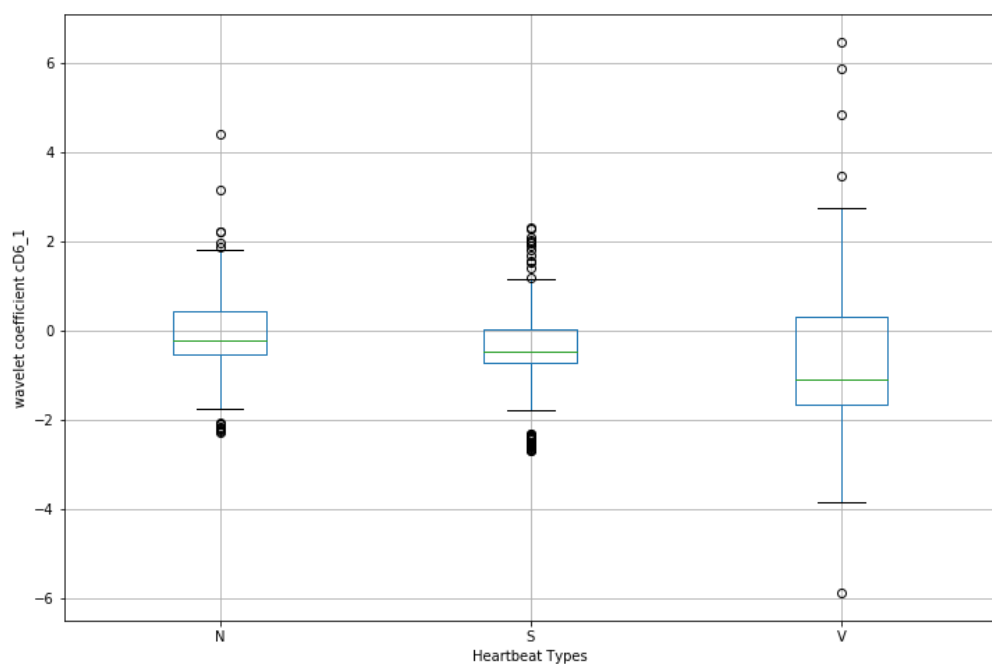



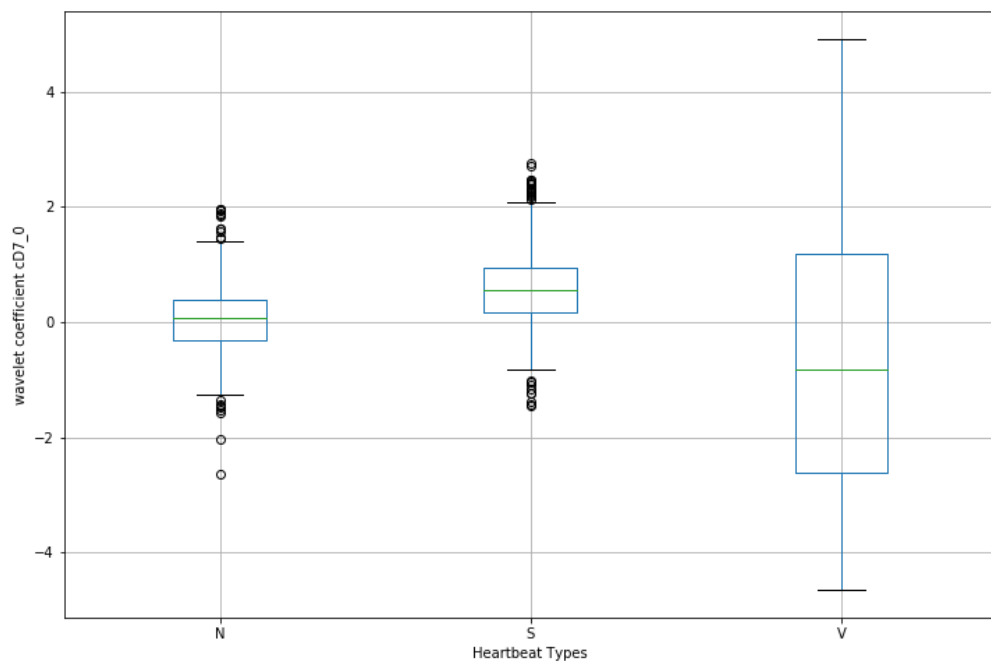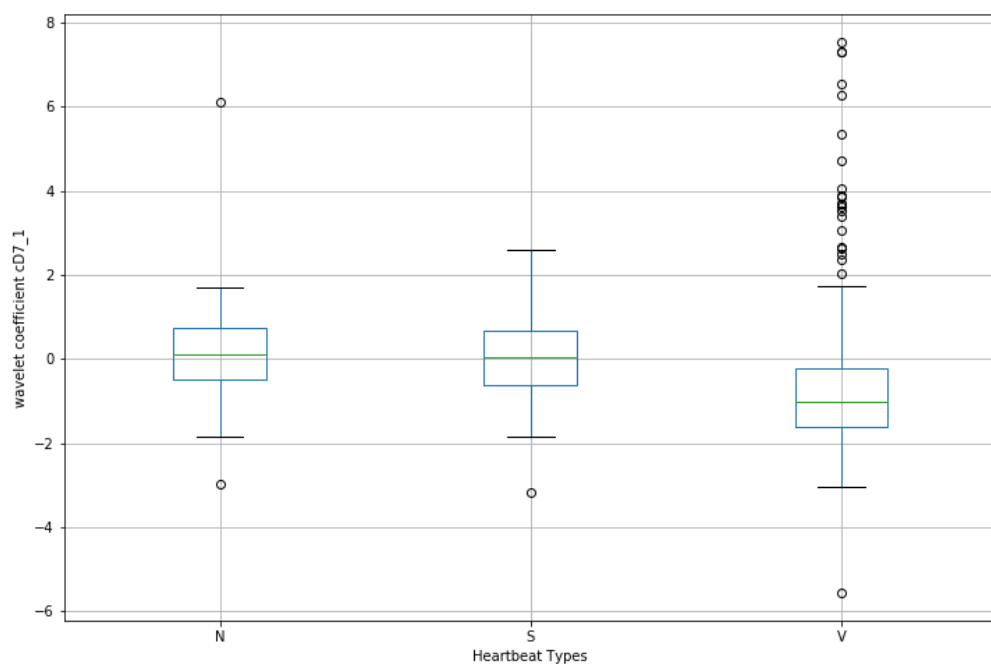

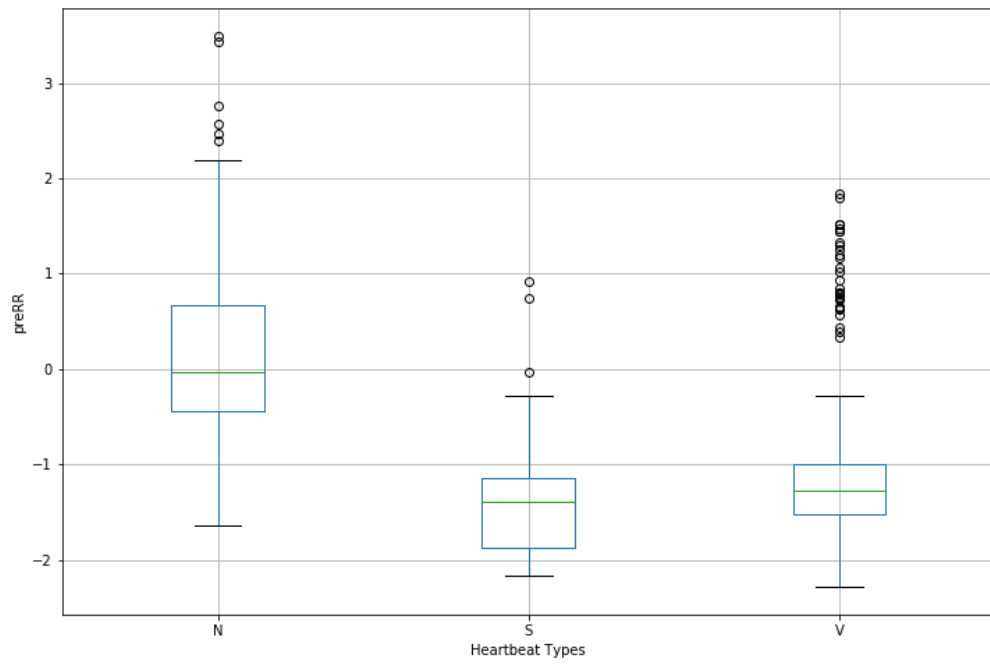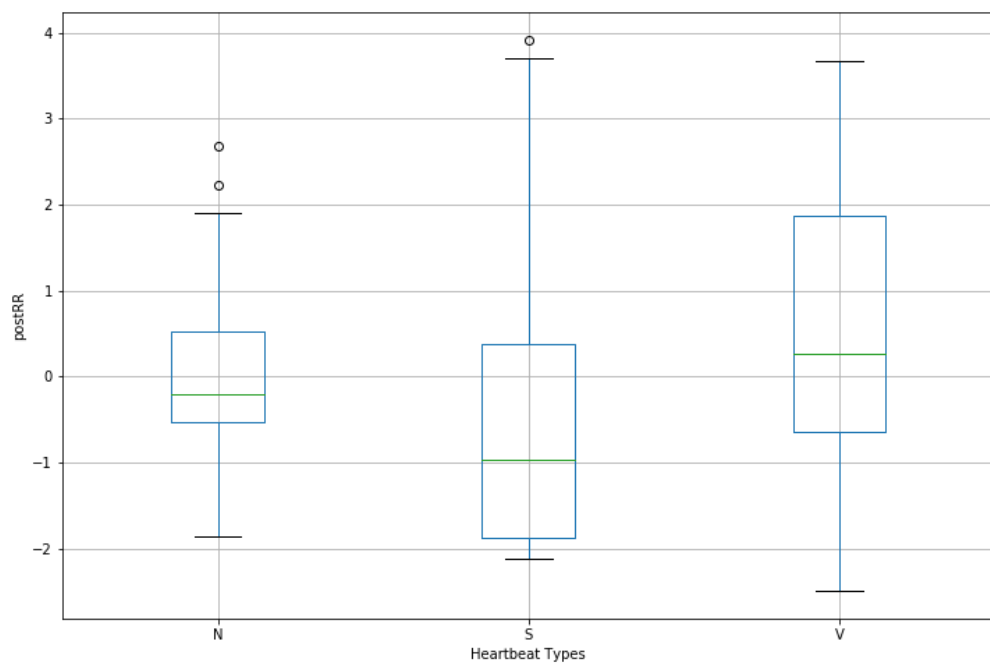

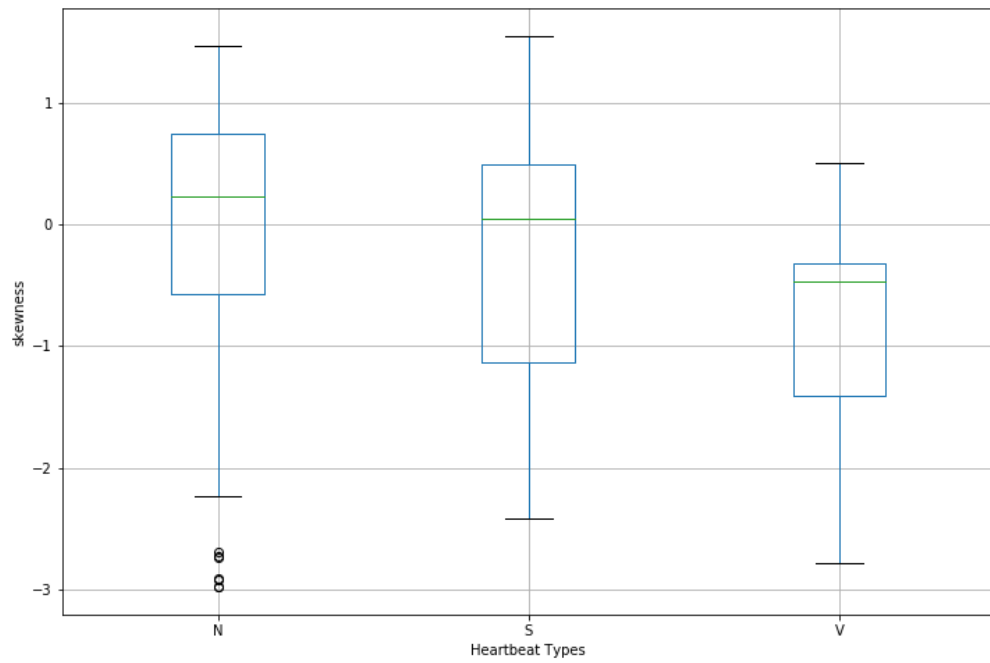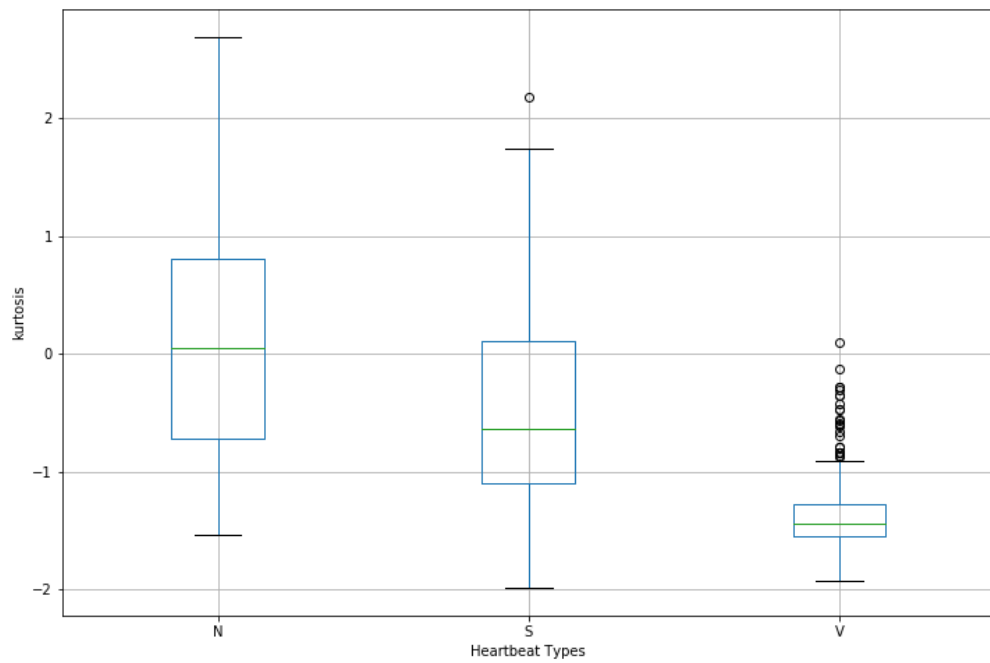

Supplement: S2 File — This file contains boxplots for all features. (PDF) [file pone.0206593.s002.pdf]
